# Comparative Analysis of Gene Expression in Virulent and Attenuated Strains of Infectious Bronchitis Virus at Subcodon Resolution

**DOI:** 10.1128/JVI.00714-19

**Published:** 2019-08-28

**Authors:** Adam M. Dinan, Sarah Keep, Erica Bickerton, Paul Britton, Andrew E. Firth, Ian Brierley

**Affiliations:** a Division of Virology, Department of Pathology, University of Cambridge, Cambridge, United Kingdom; b The Pirbright Institute, Woking, Surrey, United Kingdom; University of Texas Southwestern Medical Center

**Keywords:** RNA virus, RNASeq, avian coronavirus, differential gene expression, ribosome profiling, translation

## Abstract

Like all coronaviruses, avian infectious bronchitis virus (IBV) possesses a long, single-stranded, positive-sense RNA genome (∼27 kb) and has a complex replication strategy that includes the production of a nested set of subgenomic mRNAs (sgmRNAs). Here, we used whole-transcriptome sequencing (RNASeq) and ribosome profiling (RiboSeq) to delineate gene expression in the IBV M41-CK and Beau-R strains at subcodon resolution. RNASeq facilitated a comparative analysis of viral RNA synthesis and revealed two novel transcription junction sites in the attenuated Beau-R strain, one of which would generate a sgmRNA encoding a ribosomally occupied open reading frame (dORF) located downstream of the nucleocapsid coding region. RiboSeq permitted quantification of the translational efficiency of virus gene expression and identified, for the first time, sites of ribosomal pausing on the genome. Quantification of reads flanking the programmed ribosomal frameshifting (PRF) signal at the genomic RNA ORF1a/ORF1b junction revealed that PRF in IBV is highly efficient (33 to 40%). Triplet phasing of RiboSeq data allowed precise determination of reading frames and revealed the translation of two ORFs (ORF4b and ORF4c on sgmRNA IR), which are widely conserved across IBV isolates. Analysis of differential gene expression in infected primary chick kidney cells indicated that the host cell response to IBV occurs primarily at the level of transcription, with global upregulation of immune-related mRNA transcripts following infection and comparatively modest changes in the translation efficiencies of host genes. Cellular genes and gene networks differentially expressed during virus infection were also identified, giving insights into the host cell response to IBV infection.

**IMPORTANCE** IBV is a major avian pathogen and presents a substantial economic burden to the poultry industry. Improved vaccination strategies are urgently needed to curb the global spread of this virus, and the development of suitable vaccine candidates will be aided by an improved understanding of IBV molecular biology. Our high-resolution data have enabled a precise study of transcription and translation in cells infected with both pathogenic and attenuated forms of IBV and expand our understanding of gammacoronaviral gene expression. We demonstrate that gene expression shows considerable intraspecies variation, with single nucleotide polymorphisms being associated with altered production of sgmRNA transcripts, and our RiboSeq data sets enabled us to uncover novel ribosomally occupied ORFs in both strains. The numerous cellular genes and gene networks found to be differentially expressed during virus infection provide insights into the host cell response to IBV infection.

## Abstract (precis)

IBV is a major avian pathogen and presents a substantial economic burden to the poultry industry. Improved vaccination strategies are urgently needed to curb the global spread of this virus, and the development of suitable vaccine candidates will be aided by an improved understanding of IBV molecular biology. Our high-resolution data have enabled a precise study of transcription and translation in cells infected with both pathogenic and attenuated forms of IBV and expand our understanding of gammacoronaviral gene expression. We demonstrate that gene expression shows considerable intraspecies variation, with single nucleotide polymorphisms being associated with altered production of sgmRNA transcripts, and our RiboSeq data sets enabled us to uncover novel ribosomally occupied ORFs in both strains. The numerous cellular genes and gene networks found to be differentially expressed during virus infection provide insights into the host cell response to IBV infection.

## INTRODUCTION

**FIG 1:**
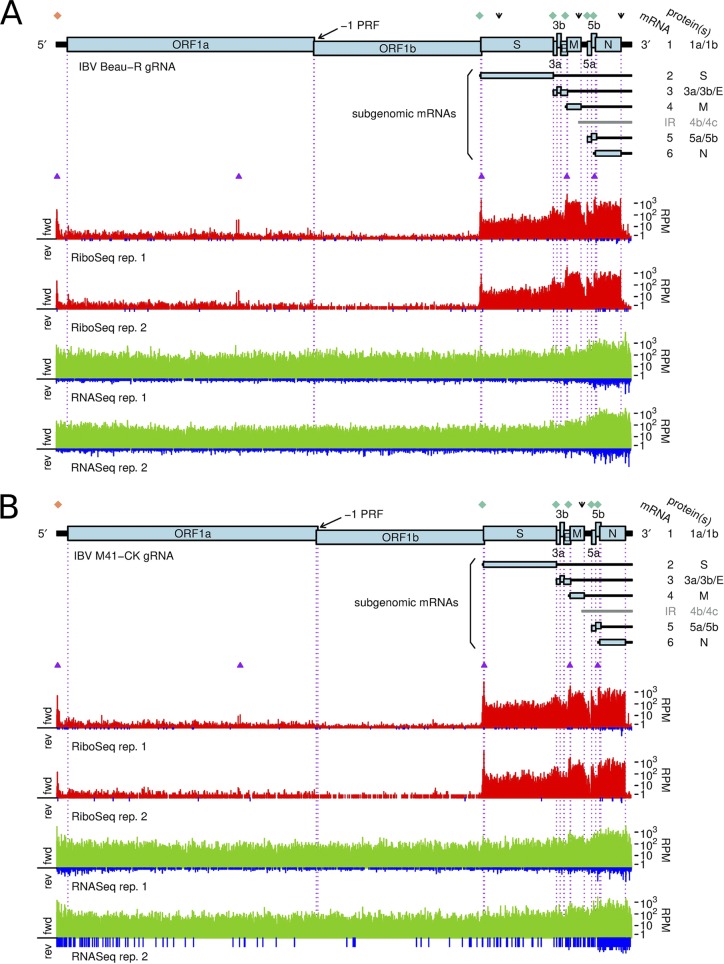
Structure and read coverage of the IBV Beau-R (A) and IBV M41-CK (B) genomes. Coverage in the RiboSeq (red) and RNASeq (green) libraries is plotted on a logarithmic scale, with negative-sense reads being shown in blue. The histograms show the positions of the 5′ ends of the reads. The 5′ two-thirds of the IBV gRNA contains two large ORFs (ORF1a and ORF1b) encoding pp1a and pp1b, respectively. Translation of the latter requires −1 programmed ribosomal frameshifting (PRF) at the indicated site. A nested set of 3′-coterminal sgmRNAs is produced during infection. Diamond symbols show the locations of canonical TRSs at which discontinuous transcription occurs (TRS-L in orange and TRS-B in green). Downward arrows indicate the positions of noncanonical TRSs discussed in this work. Purple triangles indicate sites of ribosomal pausing (see the text). fwd, forward; rev, reverse; rep., repeat; RPM, reads per million mapped reads.

Avian infectious bronchitis virus (IBV) is a member of the genus *Gammacoronavirus* (family *Coronaviridae*, order *Nidovirales*) and a pathogen of domestic fowl (1). IBV infects primarily the epithelial cells of upper and lower respiratory tract tissues, though infections can also spread to the alimentary canal, as well as to the kidneys, testes, and oviduct (2). The monopartite, polycistronic genomic RNA (gRNA) of IBV is approximately 27 kb in length, and like the gRNAs of other coronaviruses, it is 5′ methyl capped and 3′ polyadenylated (3). Two large open reading frames (ORFs)—ORF1a and ORF1b—are situated within the 5′ proximal two-thirds of the genome. Translation of the former yields a ca. 3,950-amino-acid (aa) polyprotein (pp1a), whereas translation of the latter requires −1 programmed ribosomal frameshifting (PRF) (4, 5), giving rise to a ca. 6,630-aa polyprotein (pp1ab). These polyproteins are cleaved to yield the components of the membrane-bound replication-transcription complex (RTC) (6–8). A feature of coronavirus replication is the synthesis of a nested, 3′-coterminal set of subgenomic mRNAs (sgmRNAs) encoding the viral structural and accessory proteins. The 5′ end of each sgmRNA comprises a 56-nucleotide (nt) sequence derived from the 5′ end of the genome, the so-called leader sequence (9, 10). Incorporation of the leader occurs as a result of polymerase hopping—or discontinuous transcription—during negative-strand synthesis. When the RTC encounters specific body transcription regulatory sequences (TRS-Bs), the nascent negative strand can re-pair with a closely homologous leader TRS (TRS-L) at the 3′ end of the leader, after which the viral polymerase completes negative-strand synthesis using the leader as the template (Fig. 1A, diamond symbols) (8–14). Subsequently, the RTC synthesizes positive-strand copies of the negative-strand genomic and sgmRNAs.

Among the best-characterized strains of IBV are those belonging to the Massachusetts serotype, which includes the virulent Massachusetts 41 (M41-CK) (15) isolate and the laboratory-attenuated Beau-R variant (16). While M41-CK is restricted to growth in primary chicken cells, Beau-R is capable of replicating in both avian and nonavian cell lines, including Vero (African green monkey kidney-derived) and baby hamster kidney cells (17–20). Polymorphisms in the spike (S) glycoprotein subunit 2 (S2), which spans the viral membrane, have been shown to be responsible for this variation in host cell tropism (21). Moreover, the S protein of M41-CK—but not that of Beau-R—elicits an immunoprotective response *in vivo*, although recombinant transfer of the protein from the former to the latter does not restore pathogenicity (22). The extent to which these strains diverge in terms of virus gene expression or in terms of host cell gene expression in response to infection has not been investigated in detail.

The advent of high-throughput sequencing techniques offers a means to monitor viral gene expression at unprecedented resolution (23–27). Here, we performed deep sequencing of ribosome-protected fragments (RPFs)—known as ribosome profiling (RiboSeq)—in tandem with whole-transcriptome sequencing (RNASeq) on total RNA extracts from primary chicken kidney (CK) cells infected with the Beau-R and M41-CK strains of IBV.

(This article was submitted to an online preprint archive [28].)

## RESULTS

### RiboSeq and RNASeq data quality.

RiboSeq and RNASeq libraries were prepared from two biological repeats each of Beau-R-infected, M41-CK-infected, and mock-infected cells. Infections were at a high multiplicity (multiplicity of infection [MOI] = ∼3), and cells were processed at 24 h postinfection (p.i.). An average of 1,156,819 RPFs and 1,727,024 RNASeq reads were mapped to viral gRNA in the virus-infected RiboSeq libraries (see Table S1 in the supplemental material). The RNASeq read coverage in the library derived from the second biological repeat of M41-CK-infected cells was lower than that in the other libraries due to technical losses. However, 106,741 reads were mapped to the forward strand of the viral gRNA in this case, corresponding to a coverage of approximately 3.8-fold, and these reads were generally evenly distributed along the gRNA; hence, the sequencing depth in this replicate was deemed sufficient for further analysis. The vast majority of RPFs mapping to viral and host protein-coding regions were between 27 and 29 nt in length (Fig. S1), consistent with the size of the RNA fragment protected by translating eukaryotic ribosomes from digestion by RNase I (29). The length distributions of the RNASeq reads were much broader, in line with the size of the gel slice excised for sequencing of fragmented RNA. The RPF length was strongly related to the RPF phase relative to the reading frame of the associated coding region: 27-nt RPFs were primarily in the +1 phase, whereas 28- and 29-nt RPFs were primarily in the 0 phase (Fig. S2). As expected, RNASeq reads were far more evenly split over the three phases, with a slight bias toward phase 0 (Fig. S3), which may reflect codon usage bias, such as a preference for the use of RNY codons (25, 30, 31), compounded by adaptor-ligation bias during library preparation. A meta-analysis of host mRNA coding regions showed that the depth of coverage of RiboSeq 5′ read ends increased substantially 12 nt upstream of the AUG (initiation) codon for RPFs in phase 0 (generally 28- and 29-nt RPFs) and 11 nt upstream of the AUG codon for RPFs in phase +1 (generally 27-nt RPFs) (Fig. S4 and S5). This indicates that the ribosomal P site is situated at an offset of 11 and 12 nt from the 5′ ends of RPFs for 27-nt and 28-/29-nt reads, respectively (23). Peaks in RNASeq 5′ read end coverage were seen at the A of initiation (AUG) codons and at the middle nucleotide of termination (UNN) codons, respectively (Fig. S6 and S7), and are considered an artifact of ligation bias.

Figure 1 illustrates the RiboSeq (red) and RNASeq (green) read coverage of the Beau-R (Fig. 1A) and M41-CK (Fig. 1B) genomes. In both cases, the density of the RPFs was considerably higher toward the 3′ ends of the gRNA, consistent with production of the 3′-coterminal nested set of sgmRNAs. In contrast, RiboSeq coverage of the ORF1a and ORF1b coding sequences was relatively low, reflecting the fact that a substantial proportion of newly synthesized gRNA (but not sgmRNA) transcripts is likely destined for packaging rather than translation (32). On average, negative-sense RNASeq reads were present at 0.28% of the level of positive-sense reads, indicating a ratio of positive-stranded RNA/negative-stranded RNA of ∼350:1 at 24 h p.i., a ratio similar to that seen in ribosome profiling studies of the betacoronavirus mouse hepatitis virus (MHV) (25). Negative-sense RPFs, which may represent contamination from ribonucleoprotein complexes (25), were observed, but at a low abundance (0.03% of the level of positive-sense RPFs).

### Virus transcription: sequence divergence associated with IBV strain-specific TRS usage.

**FIG 2:**
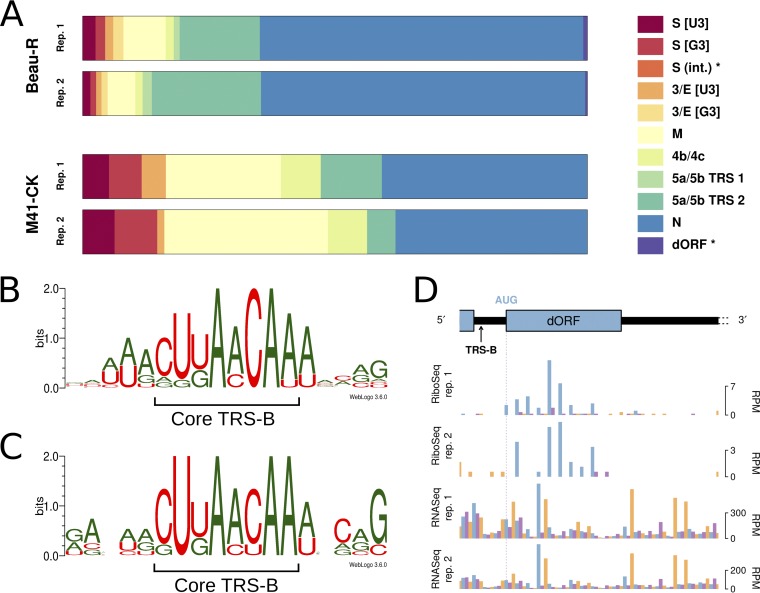
(A) Proportion of chimeric reads assigned to each of the indicated TRS junctions. Novel TRSs identified in this study are indicated with asterisks. Note that the 5a/5b TRS 1 and the dORF TRS are present in IBV Beau-R only. (B) Sequence logo depicting nucleotides surrounding the identified TRS-B sites in IBV Beau-R. (C) Sequence logo equivalent to that described in the legend to panel B for IBV M41-CK. (D) RiboSeq and RNASeq coverage of the IBV Beau-R dORF. A +12-nt offset is applied to the 5′ ends of all reads, to approximate the position of the ribosomal P site in RiboSeq libraries (and to make the RNASeq coverage directly comparable). Reads whose 5′ ends map to the first, second, or third positions of codons are indicated in blue, purple, and orange, respectively, and ORFs are colored according to the frame in which they are encoded. The location of the novel TRS-B sequence, which begins 2 nt 3′ of the N gene termination codon, is indicated with an arrow. int, internal TRS.

The density of RNASeq reads mapping to a given sgmRNA represents the cumulative sum of reads derived from the gRNA and those derived from the overlapping portions of other subgenomic transcripts (Fig. 1). Therefore, to estimate the abundance of individual sgmRNAs, two independent approaches were used. First, we decumulated the raw RNASeq read densities mapping to inter-TRS regions, by subtracting the density of the 5′-adjacent inter-TRS region in each case (25). Second, the abundances of chimeric RNASeq reads spanning TRS junctions were quantified, by identifying unmapped reads containing an 11-nt sequence derived from the leader region 5′ adjacent to the TRS-L (UAGAUUUUUAA, nt 46 to 56 in Beau-R; UAGAUUUCCAA, nt 46 to 56 in M41-CK) and including at least 16 nt 3′ of this query. Chimeric reads were assigned to specific genomic loci based on the sequences 3′ of the TRS in each case (Fig. 2A; Table S2). Overall, the chimeric read abundances for sgmRNAs were significantly correlated with the corresponding decumulated RNASeq densities (*P < *0.01 in both cases) (Fig. S8). The sequence logos in Fig. 2B and C illustrate the diversity of nucleotides found at TRS-B sites identified in this study (including the novel sites discussed below) in Beau-R and M41-CK, respectively. The core region of similarity to the TRS-L motif (CUUAACAA) was typically flanked by a 3′ adenine (A) or uracil (U) residue, and a preference for A/U residues was also seen immediately upstream of the core sequence. These flanking residues may facilitate template switching by lowering the free energy of anti-TRS-B/TRS-B duplex disassociation, since the TRS-L is also located in an AU-rich region (14).

Notably, the A nucleotides at positions 4 and 7 of the core motif are the only invariant residues. In both Beau-R and M41-CK, the TRS-B sequences associated with the S gene contain G residues at the third positions (CUGAACAA), in contrast to the TRS-L, which has a U at this position (CUUAACAA) (Table S2). Chimeric reads assigned to this gene were found to contain either a U or a G residue at position 3 (denoted S [U3] and S [G3], respectively, in Fig. 2A; Table S2), with a G being more common in M41-CK (7.5% of reads compared with 5.8%, on average) and a U being more common in Beau-R (2.1% of reads compared with 1.5%, on average) (Table S2). These data indicate that the exact position at which discontinuous transcription occurs within a given TRS is subject to some variation, with either the TRS-L or the TRS-B templating the third residue. Similarly, the TRS-B for the 3a/3b/E genes diverges at the third position between Beau-R (CUGAACAA; nt 23825 to 23832) and M41-CK (CUUAACAA; nt 23832 to 23839), with the latter matching the TRS-L sequence exactly. In this case, we found that Beau-R-derived chimeric reads could contain either a U (denoted 3/E [U3]) or a G (denoted 3/E [G3]), with the G residue being slightly more common (1.6% versus 1.2%, respectively, on average), whereas M41-CK-derived reads contained only the U residue (Fig. 2A; Table S2).

In agreement with a previous report (33), we found that the 3′-most of two adjacent canonical TRS-B sequences (both CUUAACAA; nt 25460 to 25467 and nt 25471 to 25478, labeled 5a/5b TRS 1 and 5a/5b TRS 2, respectively, in Fig. 2A) within the 30-nt region upstream of genes 5a/5b was preferentially utilized in IBV Beau-R, accounting for 18.8% of chimeric reads, on average, compared with 1.5% for the 5′-most TRS-B. Interestingly, more chimeric reads were assigned to the noncanonical TRS-B associated with genes 4b/4c (34), which has a low homology to the TRS-L (Table S2), than to the first of these 5a/5b-associated TRSs in IBV Beau-R, emphasizing the importance of the genomic context in facilitating discontinuous transcription (Fig. 2B and C) (14). Only one of the two 5a/5b TRSs (TRS 2) was found in the IBV M41-CK genome (Fig. 2A).

### Novel TRS in IBV Beau-R.

Two additional noncanonical leader/body chimeras, both specific to the Beau-R strain, were identified (Table S2). The more abundant of these (0.6% of chimeric reads) mapped to a position immediately downstream of the IBV Beau-R N gene termination codon within the 3′ untranslated region (UTR). Chimeric reads derived from this site contained the sequence CUUAACAU, the last 6 nt of which could have been templated by the genomic (TRS-B) sequence (UAACAU, nt 27104 to 27109). There was an AUG-initiated downstream ORF (dORF) in Beau-R beginning 2 nt 3′ of this TRS, which comprised 11 codons (nt 27111 to nt 27143). Inspection of our RiboSeq libraries showed that the dORF is ribosomally occupied (Fig. 2D). Such AUG-initiated dORFs are present immediately 3′ of the N genes in most IBV strains and related avian coronaviruses, including turkey coronavirus (TCoV), goose coronavirus (35, 36), and pigeon coronavirus (35), but this region appears to have been deleted in the IBV M41-CK lineage, and M41-CK also lacks the TRS-B downstream of the N gene (UAAAAU, nt 27156 to 27161).

The second novel chimeric sequence identified in RNASeq libraries maps to a TRS-B (CUUACCAA) within the coding region of the S gene in Beau-R (nt 21242 to 21249). This is consistent with the previous detection of an sgmRNA of appropriate length via Northern blot analysis (34). While the core sequence of the TRS-B in this case is conserved in M41-CK, there is a single nucleotide (A-to-C) polymorphism located 4 nt downstream in the 3′ flanking region, which may contribute to its lack of utilization in this strain (Table S2).

### Virus translation: direct measurement of –1 PRF between ORF1a and ORF1b.

**FIG 3:**
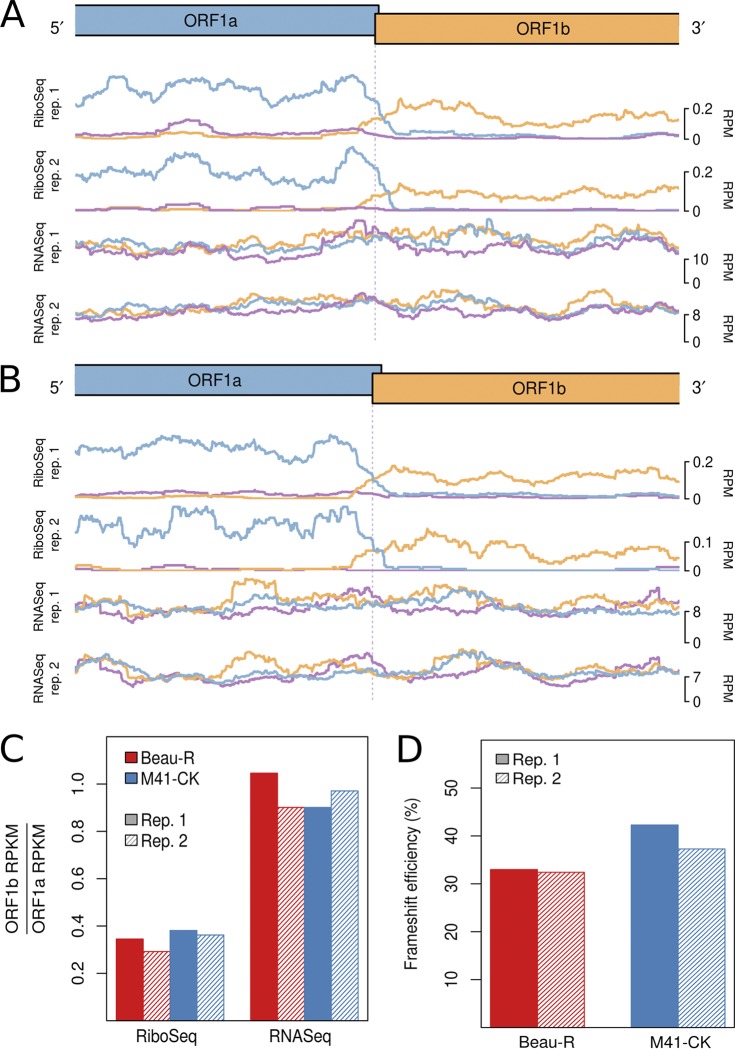
RiboSeq and RNASeq coverage proximal to the junction between ORF1a and ORF1b for IBV Beau-R (A) and IBV M41-CK (B). The last 2,500 nt of ORF1a and the first 2,500 nt of ORF1b are shown. Coverage is normalized to reads per million mapped reads (RPM), determined using the sum of total virus RNA plus total host RefSeq mRNA (positive sense reads only) as the denominator and smoothed with a 121-codon sliding window. Reads in phase 0, +1, and +2 relative to ORF1a are shown in blue, purple, and orange, respectively; and ORFs are colored according to the frame in which they are encoded. (C) Ratios of ORF1b to ORF1a read density expressed as reads per kilobase per million mapped reads (RPKM). RPKM values exclude the 150-nt regions downstream of the ORF1a initiation codon, upstream of the ORF1b termination codon, and on either side of the frameshift site. (D) Frameshifting efficiencies calculated using the values plotted in panel C.

Ribosome profiling of eukaryotic systems typically has the characteristic that mappings of the 5′ end positions of RPFs to coding sequences reflect the triplet periodicity of genetic decoding. A clear phase transition is evident in the RiboSeq libraries at the junction of ORF1a and ORF1b, where frameshifting of a proportion of ribosomes from the former ORF into the latter occurs (Fig. 3A and B). The mean normalized ratios of ORF1b to ORF1a RiboSeq density were 0.32 and 0.37 in IBV Beau-R and IBV M41-CK, respectively, while the corresponding RNASeq ratios were 0.97 and 0.94, respectively (Fig. 3C). Thus, on average, 33% of ribosomes in Beau-R and 39% in M41-CK undergo –1 PRF prior to reaching the ORF1a termination codon (Fig. 3D). These values are very similar to those measured in *in vitro* PRF assays (4, 5), and alongside related profiling studies of MHV (25), this indicates that coronaviruses exhibit highly efficient PRF both *in vitro* and in the context of the infected cell.

### Ribosomal occupancy of ORF4b and ORF4c.

**FIG 4:**
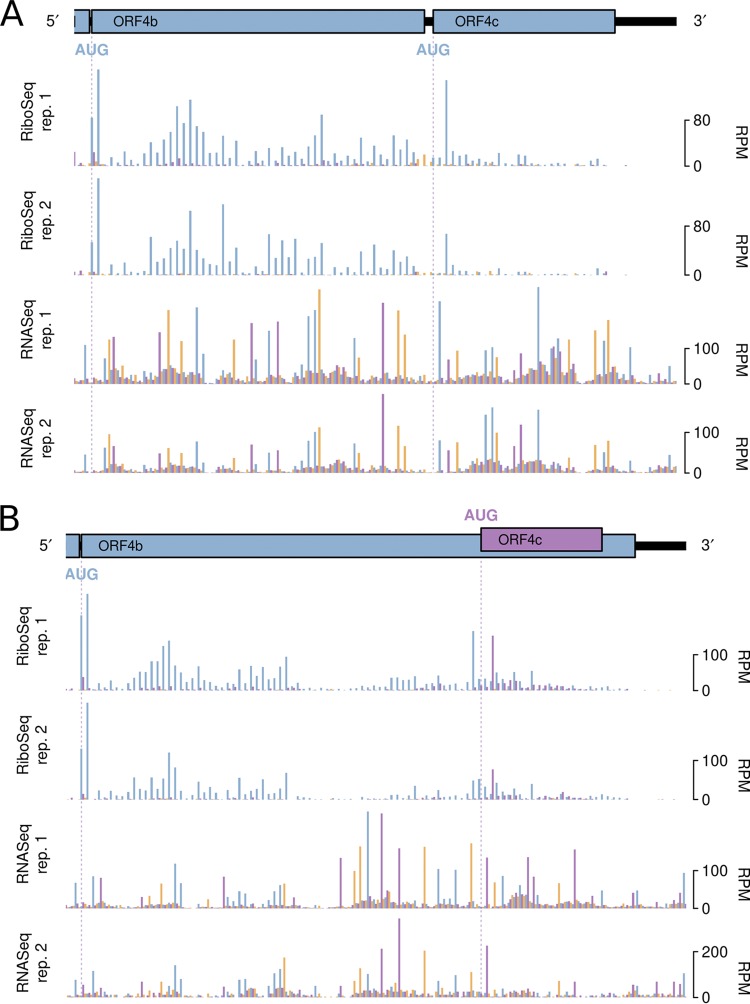
RiboSeq and RNASeq coverage of ORF4b and ORF4c in IBV Beau-R (A) and IBV M41-CK (B). Coverage is expressed as reads per million mapped reads (RPM). Reads in phase 0, +1, and +2 relative to ORF4b are shown in blue, purple, and orange, respectively; and ORFs are colored according to the frame in which they are encoded.

Situated between the M and 5a genes in Beau-R and M41-CK is a >300-nt ostensibly intergenic region (IR) (Fig. 1). No protein-coding genes are annotated here, but two putative AUG-initiated ORFs are present in each virus and are referred to as ORF4b and ORF4c, after their homologs in turkey coronavirus (TCoV) (37, 38) and in the genomes of most IBV isolates (39). The putative ORF4b genes of Beau-R and M41-CK are encoded by nt 25183 to 25335 (50 codons) and nt 25190 to 25474 (94 codons), respectively, of the gRNA, whereas the ORF4c genes are encoded by nt 25339 to 25422 (27 codons) and nt 25395 to 25457 (20 codons), respectively (Fig. S9). Thus, in Beau-R, the two ORFs are separated by a 3-nt spacer region and are in the same reading frame (Fig. 4A), whereas in M41-CK, ORF4c is located entirely within the ORF4b gene and is in the +1 phase (Fig. 4B). Inspection of the ribosomal profiling data sets reveals substantial RPF coverage of both ORF4b and ORF4c, providing the first clear illustration that ORFs 4b and 4c are ribosomally occupied (Fig. 4). Visualization of ORF4c translation in M41-CK was facilitated by good phasing in the data sets, allowing expression of both ORF4b and ORF4c to be visualized (as both blue and orange RPF peaks in the overlap region in Fig. 4). Previous work (34) has shown that a noncanonical TRS-B sequence—situated approximately 100 nt upstream of the M gene termination codon—facilitates production of an sgmRNA (IR) that harbors ORF4b at its 5′ end, and this TRS-B was also identified in our RNASeq data.

### Translation efficiencies of IBV genes.

**FIG 5:**
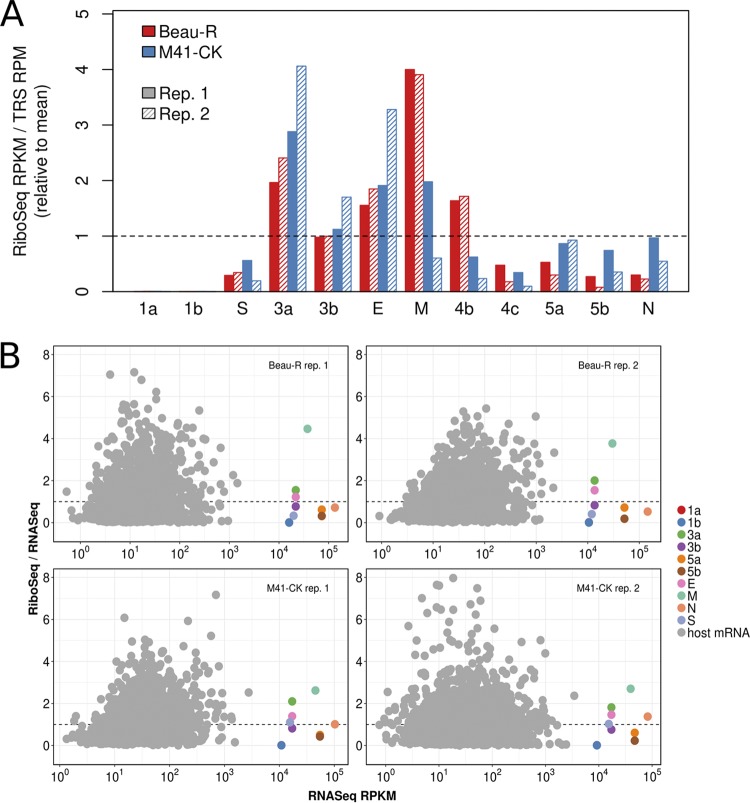
(A) Translation efficiencies (TEs) of virus ORFs, as calculated using the relative abundances (reads per million [RPM]) of chimeric TRS-spanning RNASeq reads. Values shown are relative to the mean efficiency per TRS (the TEs of virus ORFs calculated using decumulated RNASeq densities are shown in Fig. S10 in the supplemental material). (B) Comparison of host and virus translation efficiencies. The TEs of virus ORFs were calculated using decumulated RNASeq densities, as described in the text. Host mRNA TEs are based on the ratio (after normalization for library size) of all RiboSeq or RNASeq reads mapping to any annotated coding region of any splice form of a given gene. Host data are shown only for genes with ≥50 RNASeq coding-region reads, on average, across samples (prior to normalization for library size). Horizontal dashed lines indicate the mean values for host cell genes. Note that the points for 1a and 1b overlap.

To estimate the translational efficiency (TE) of virus genes, we summed RPFs whose 5′ end mapped in phase between the first nucleotide of the initiation codon and 30 nt 5′ of the termination codon, thereby excluding RPFs derived from ribosomes paused during initiation or termination (the raw ribosome footprint data are provided in Table S3). The TE of each ORF was measured as the quotient of the RPF density and the abundance of the corresponding mRNA, with separate calculations being performed using TRS chimeric reads counts and the decumulated RNASeq densities (Fig. 5A and Fig. S10, respectively). In the case of ORF4b and ORF4c, transcript abundance could not be accurately deduced via the RNASeq decumulation procedure, because the significantly lower level of expression of the 4b/4c transcript relative to that of the 5′-adjacent M gene (Fig. 1) was associated with a proportionate increase in the level of noise. Similarly, as a result of the high abundance of gRNA relative to the sgmRNA encoding S, the decumulated RNASeq density for the latter is likely to be poorly estimated, and therefore, the TE value for S calculated using the chimeric read count is likely to be more accurate. From this analysis, it was observed that the 4b gene is more efficiently translated than the 4c gene, a trend also observed for the accessory genes 3a/3b and 5a/5b (Fig. 5A). This is consistent with the likely requirement for leaky scanning to access the downstream ORF on each sgmRNA (see Discussion). Surprisingly, despite the fact that the nucleocapsid (N) protein is an abundant viral protein, it was not found to be efficiently translated relative to the other structural proteins, regardless of the approach used to estimate transcript abundance (Fig. 5A; Fig. S10). In the case of the ORF1a and ORF1b genes, a large proportion of the genomic RNA is expected to be destined for packaging rather than translation, as mentioned above, and this probably explains the low TE values calculated for these genes (Fig. 5A; Fig. S10). Additionally, the short length of the dORF precluded an accurate assessment of its translation efficiency. Figure 5B compares the translation efficiencies of virus and host coding DNA sequences (CDSs), with the former being calculated using decumulated RNASeq densities. The latter are calculated on a per gene (rather than per transcript) basis, using RNASeq and RiboSeq reads contained entirely within annotated CDS regions (i.e., excluding 5′ and 3′ UTRs and also RPFs accumulating at or near initiation or termination sites), and, like the virus values, are expressed relative to the mean levels for the cell (due to normalization by library size). The analysis shows that the virus translation efficiencies fall within the general range of those of host genes and indicates that virus transcripts are not preferentially translated during virus infection. Instead, the massive production of virus proteins (in particular, the N protein) is achieved through high levels of transcription.

### Ribosomal pauses during IBV genome translation.

**FIG 6:**
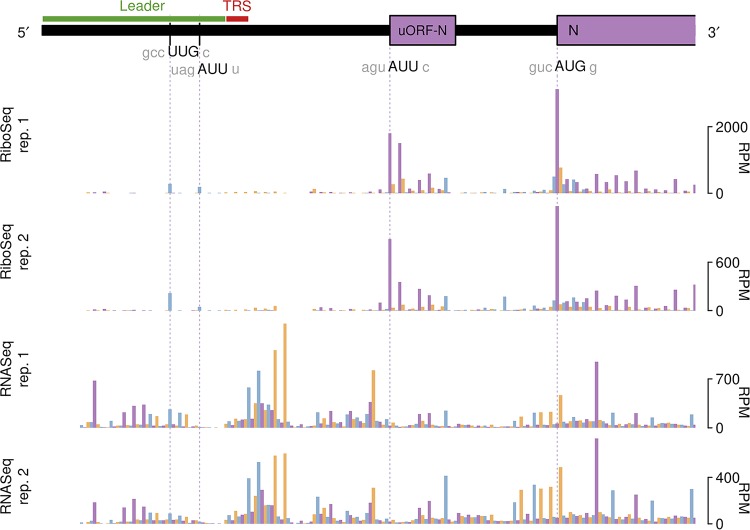
RiboSeq and RNASeq coverage of sgmRNA N in IBV Beau-R. Coverage is expressed as reads per million mapped reads (RPM). Reads in phase 0, +1, and +2 relative to N are shown in purple, orange and blue, respectively; and ORFs are colored according to the frame in which they are encoded.

Inspection of the profiling data sets revealed a number of genomic locations where RPFs accumulated to a much higher level than at neighboring sites, indicative of ribosomal pausing. As such pauses may have biological significance, we first sought to discount those that may have arisen artefactually. The known translation initiation sites in the virus genome generally showed high ribosome occupancy, but as the infected cells were treated with cycloheximide (CHX) prior to lysis to freeze ribosomes onto the mRNA, these pauses are likely to be overrepresented, as ribosomes can accumulate at start codons during the CHX treatment period (40). Fluctuations in RPF density can also occur as a result of nuclease, ligation, and PCR biases during library construction. As the latter two biases can also occur during RNASeq library generation, we also discounted any pauses that had an obvious counterpart in RNASeq data sets. With these criteria, we identified five obvious sites of ribosomal pausing conserved in Beau-R and M41-CK, one in the 5′ UTR and four within the coding region (indicated in Fig. 1, purple triangles; Table 1). Pauses in 5′ UTRs can represent ribosomes initiating at upstream ORFs (uORFs), although in both Beau-R and M41-CK, the P site of the ribosome that paused over bases 28 to 56 in the 5′ UTR of the genome is on a non-AUG codon (UUG) in a weak Kozak initiation consensus. As this pause is located upstream of the TRS-L, it reflects the sum of pausing on all sgmRNAs. To view the extent of the pause in context, we remapped reads to the most abundant sgmRNA, i.e., that of the N gene (Fig. 6). As can be seen, the leader pause remains clearly evident (as is a smaller pause 3 codons downstream), albeit it is smaller in magnitude than those pauses seen at an N uORF (see below) and the authentic AUG codon of the N protein. Initiation at the UUG codon would result in translation of solely a dipeptide, and, thus, the pause, if biologically relevant, may act as a regulator of downstream initiation events rather than through the encoded product. We note that an equivalent leader pause is seen in MHV (UUG codon, 1-codon ORF [25]). It is possible that pausing at this codon is potentiated by queueing of initiating ribosomes on sgmRNAs. The origins of the pauses within the coding region are enigmatic. The two adjacent pauses referred to collectively as pause 2 in Table 1 correspond to translation of a region of nonstructural protein 4 (nsP4) downstream of the membrane-spanning domains (41, 42). It is feasible that ribosomes pause here while the nascent peptide is being folded into membranes. Pauses 3 and 4 are noticeably large and correspond to ribosomes pausing soon after initiation of the S and M proteins, respectively. In the case of the former, the pause is unlikely to be linked to an interaction of the signal sequence at the N terminus of the S protein with membranes, as this peptide would still be within the exit tunnel of paused ribosomes. Pause 5 corresponds to a potential non-AUG uORF (AUU, in a reasonable context) within the N mRNA (Fig. 6).

**TABLE 1:** Ribosomal pause sites within the IBV genome

Pause	Genomic location and RPF sequence^*a*^	Nascent peptide
Pause 1 (5′ NCR^*b*^)	5′ end of genome (bases 28–56) near TRS_L, 5′-AUUACACUAGCCUUGCGCUAGAUUUUUA-3′	^c^*YISITLALR*^c^
Pause 2 (nsP4)	Two adjacent peaks within nsP4 coding region (nt ∼8660 and 8760), 5′-UUUGUUAAGCUUACUAAUGAGAUAGGU-3′ and 5′-UUGCAAGCUUGUCGUGCAUGGUUAGCU-3′	YDGNEFVGNYDLAAKSTFVIRGSEFVKLTN and KFEAYLSAYARLKYYSGTGSEQDYLQACRA
Pause 3 (S)	Large pause downstream of initiation codon of S protein (nt ∼20410), 5′-CUAGUGACUCUUUUGUGUGCACUAUGU-3′ (Beau-R)	^*d*^MLVTPLLLVTLLC (Beau-R)
Pause 4 (M)	Very large pause immediately downstream of initiation codon of the M protein (nt ∼24,500), 5′-(AUG)CCCAACGAGACAAAUUGUACUCUUGACU-3′	^*d*^MPNETNC
Pause 5 (N)	Broad pause peak centered on YLSSIPREN near end of 5b ORF (∼25,830), just upstream of N start codon ribosome stack, 5′-UACCUCUCUAGUAUUCCAAGGGAAAACU-3′	QSRTSRALSRVYLSSIP(RENL*^c^)

a Underlined characters signify the codon/amino acid of the ribosomal P-site tRNA.

b NCR, noncoding region.

c *, in-frame stop codon.

d Initiator methionine.

It is noteworthy that in our analysis of ribosomal pause sites, we did not see pausing at the AUG of the previously described 11-amino-acid uORF of the genomic mRNA (AUG at nt 131 to 133 [43]), and indeed, there were few reads on the uORF itself, indicating that it is not heavily translated. Further, no pausing was observed at the PRF site at the ORF1a/ORF1b overlap.

### Differential expression of host genes in response to IBV infection.

**FIG 7:**
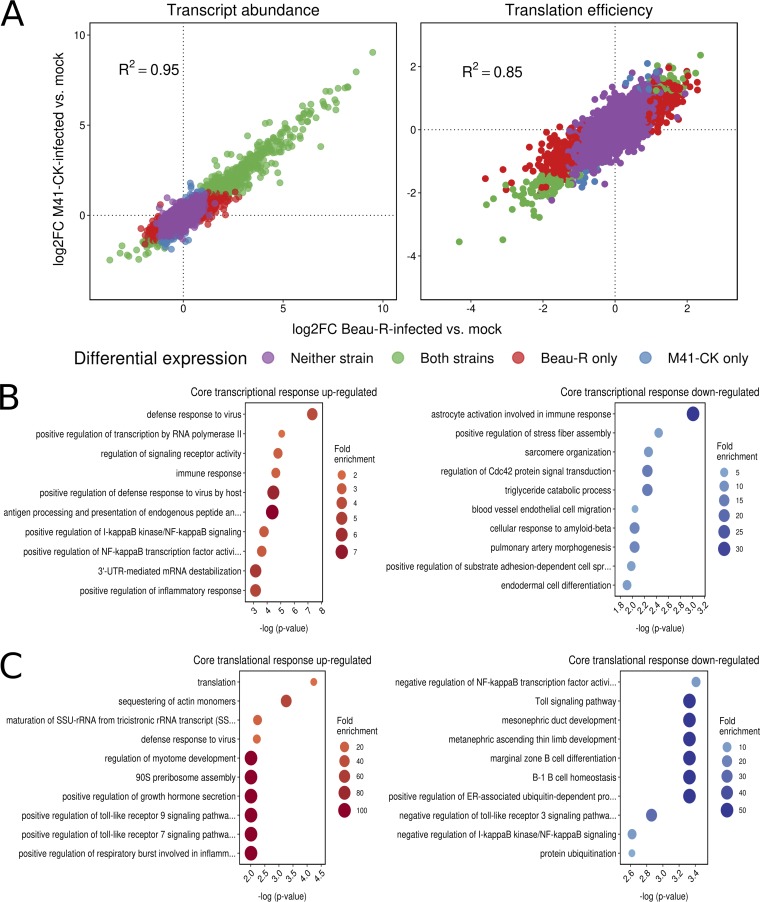
(A) Log_2_ fold changes (log_2_FC) in host transcript abundance and translation efficiency in infected cells relative to mock-infected cells. In both cases, a high degree of correlation between the log_2_FC values in Beau-R-infected samples (*x*-axes) and M41-CK-infected samples (*y*-axes) was observed, with transcript abundances being skewed toward positive log_2_FC values. (B) The 10 most significantly enriched GO terms among commonly upregulated (left) and commonly downregulated (right) genes at the level of transcription. (C) The 10 most significantly enriched GO terms among commonly upregulated (left) and commonly downregulated (right) genes at the level of translation efficiency. SSU, small subunit; ER, endoplasmic reticulum.

We investigated the differential transcription and translation of host genes in response to IBV infection by comparing RNA and RPF densities per coding region for infected and mock-infected samples (see Materials and Methods). Details of the genes found to be differentially expressed (DEGs; false-discovery rate [FDR] < 0.05 with multiple-testing correction using the Benjamini-Hochberg method) at the level of transcription (4,266 genes) or translation (3,627 genes), respectively, are provided in Data Sets S1 and S2. Overall, the patterns of change in host cell gene expression in response to infection were broadly similar for Beau-R and M41-CK, with positive interstrain correlations in the log_2_ fold changes (log_2_FC) in transcript abundance and TE (*R*^2^ = 0.95 and *R*^2^ = 0.85, respectively; *P* values for both, <2.2 × 10^−16^; Fig. 7A). Notably, the majority of differentially transcribed genes were upregulated rather than downregulated (i.e., log_2_FC > 0) for both strains (Fig. 7A, left), with 2.1-fold and 3.5-fold more upregulated than downregulated transcripts (FDR < 0.05; see Materials and Methods) being detected in Beau-R-infected cells and M41-CK-infected cells, respectively (Data Set S1). This effect was not seen at the level of translation, where there were fewer differentially expressed genes overall, and the log_2_FC values of those genes were more evenly distributed around 0, with slight skewing toward negative values (i.e., a reduced TE) (Fig. 7A, right; Data Set S2). The core host transcriptional response to the two strains involved 579 commonly upregulated and 132 commonly downregulated genes, while the core translational response consisted of 34 commonly upregulated and 79 commonly downregulated genes. Gene ontology (GO) term enrichment revealed that numerous immune-related pathways were among the most significantly enriched terms in the core response sets (Fig. 7B and C). There was also evidence of integration and coordination of responses at the transcriptional and translational levels. For example, the GO term “positive regulation of NF-κB transcription factor activity” (GO:0051092) was enriched among transcriptionally upregulated genes, whereas “negative regulation of NF-κB transcription factor activity” (GO:0032088) was enriched among translationally downregulated genes. In a direct interstrain comparison of statistically significant DEGs, we identified 51 differentially transcribed genes, 45 of which were more highly expressed in Beau-R-infected samples and 6 of which were more highly expressed in M41-CK-infected samples (Data Set S1). The most significantly enriched GO term in the former set was “regulation of signaling receptor activity” (GO:0010469), while proproliferative and antiapoptotic GO terms were also enriched (Table S4). The latter set included three heat shock protein-encoding genes, and consequently, the top enriched GO terms were related to protein refolding (Table S5). Just one gene (ENSGALG00000015358 [MYH15], encoding myosin heavy chain 15) had a significantly higher translation efficiency in M41-CK-infected samples than in Beau-R-infected samples.

**FIG 8:**
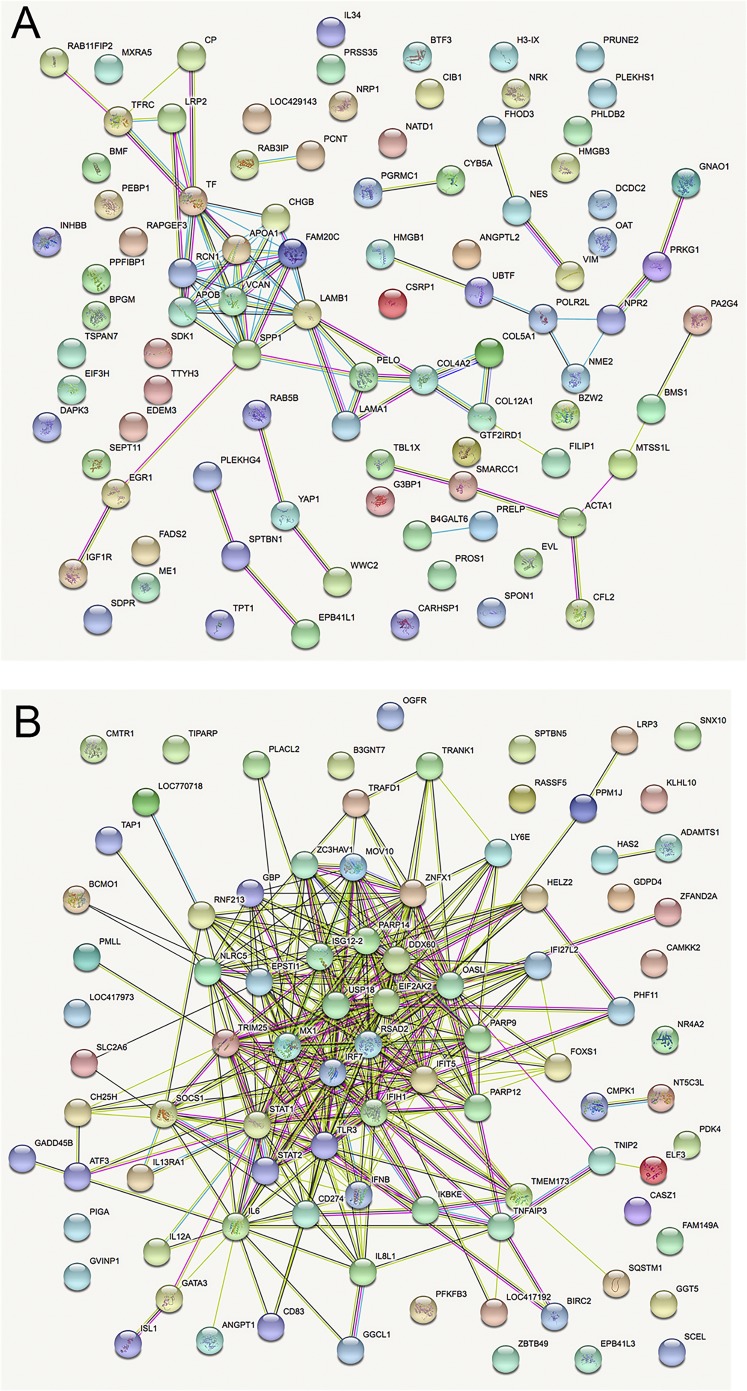
STRING analysis of the relationships between differentially expressed transcripts in comparisons of IBV M41-CK- and mock-infected cells. (A) Downregulated genes. (B) Upregulated genes. The network nodes represent the proteins encoded by the differentially expressed genes. Seven different colored lines link a number of nodes and represent seven types of evidence used in predicting associations. A red line indicates the presence of fusion evidence, a green line represents neighborhood evidence, a blue line represents co-occurrence evidence, a purple line represents experimental evidence, a yellow line represents text-mining evidence, a light blue line represents database evidence, and a black line represents coexpression evidence.

In comparisons of host gene expression between Beau-R-, M41-CK-, and mock-infected cells, the significantly differentially expressed genes (FDR < 0.05) were ranked by log_2_FC (Data Set S3), and the top 100 DEGs (or less) within each category were subjected to STRING analysis (44) to identify potential protein-protein interaction pathways (Fig. 8 and Fig. S11). A selection of the key pathways proposed and examples of the associated genes are shown in Table 2. Clear patterns of host response to virus infection were present and are discussed below. Note in the interstrain comparisons of M41-CK versus Beau-R, only the transcriptionally downregulated category had sufficient gene candidates for STRING analysis; the other three categories had a total of only seven DEGs (thus, no plots are shown in the supplemental material for these).

**TABLE 2:** STRING analysis of differential gene expression

Comparison	Parameter	Main pathway(s)	Examples in pathway(s) (FDR, log_2_FC)
Transcription			
Beau-R- vs mock-infected cells	Downregulated	FAM20C substrates	SPP1 (secreted phosphoprotein 1) (7.31 × 10^–15^, –2.72), TF (transferrin) (1.83 × 10^–13^, –3.17)
M41-CK- vs mock-infected cells	Downregulated	FAM20C substrates	SPP1 (secreted phosphoprotein 1) (6.13 × 10^–10^, –3.177), CHGB (chromogranin B) (7.42 × 10^–8^, –2.09)
Beau-R- vs mock-infected cells	Upregulated	Antiviral state, receptor signaling, cytokine interactions	RSAD2 (viperin) (3.92 × 10^–155^, 9.49), IFIT5 (interferon-induced protein with tetratricopeptide repeats 5) (4.48 × 10^–142^, 8.66)
M41-CK- vs mock-infected cells	Upregulated	Antiviral state, receptor signaling, cytokine interactions	RSAD2 (viperin) (1.80 × 10^–140^, 9.04), IFIT5 (interferon-induced protein with tetratricopeptide repeats 5) (3.01 × 10^–119^, 7.96)
M41-CK- vs Beau-R-infected cells	Downregulated	Cytokines, cytokine-receptor interactions	IL-6 (interleukin 6) (4.38 × 10^–4^, –1.51), IL-8L1 (interleukin 8-like 1) (1.38 × 10^–2^, –1.44).
M41-CK- vs Beau-R-infected cells	Upregulated	Heat shock family members^*a*^	HSPA5 (heat shock 70-kDa protein 5) (2.28 × 10^–5^, 1.74), HSP90AA1 (heat shock protein 90 alpha family class A member 1) (4.38 × 10^-–4^, 1.44)
Translation			
Beau-R- vs mock-infected cells	Downregulated	No obvious pathways identified (top two hits shown to right)	TIPARP [TCDD-inducible poly(ADP-ribose) polymerase] (8.42 × 10^–23^, –4.32), ADAMTS1 (ADAM metallopeptidase with thrombospondin type 1 motif 1) (1.0 × 10^–12^, –3.57)
M41-CK- vs mock-infected cells	Downregulated	No obvious pathways identified (top two hits shown to right)	TIPARP [TCDD-inducible poly(ADP-ribose) polymerase] (8.42 × 10^–23^, –4.32), PFKFB3 (6-phosphofructo-2-kinase/fructose-2,6-biphosphatase 3) (2.47 × 10^–13^, –3.49)
Beau-R- vs mock-infected cells	Upregulated	Antiviral response, translation, 80S ribosome, RACK1	OASL (2′-5′-oligoadenylate synthetase like) (5.08 × 10^–6^, 2.24), RPSL37 (ribosomal protein L37) (5.34 × 10^–5^, 1.78)
M41-CK- vs mock-infected cells	Upregulated	Antiviral response, 80S ribosome	OASL (2′-5′-oligoadenylate synthetase-like) (1.16 × 10^–3^, 1.79), RPS8 (ribosomal protein S8) (1.03 × 10^–2^, 1.35)
M41-CK- vs Beau-R-infected cells	Downregulated	No pathways identified	No significant genes identified
M41-CK- vs Beau-R-infected cells	Upregulated	No pathways identified	Only one significant gene identified, MYH15 (myosin heavy chain 15) (0.032, 1.77)

a Only six of the top 100 DEGs were significant in this category (see Data Set S3 in the supplemental material).

## DISCUSSION

Here, we describe the first high-resolution study of gammacoronaviral gene expression during infection of primary chick kidney cells. Analysis of RNASeq data sets through chimeric read analysis or decumulation allowed us to quantify the relative levels of viral genomic and subgenomic mRNAs and to define the sequence diversity of strain-specific TRS utilization. The predominant sgmRNA in both strains was that encoding the N protein, and between strains, the M transcript was relatively more abundant in M41-CK. In Beau-R, two novel TRSs were identified, one in the viral 3′ UTR immediately downstream of the N gene termination codon and one mapping to a TRS-B within the S gene. In the former, a short ORF (dORF), initiated 2 nt 3′ of the TRS, was present and ribosomally occupied. The potential biological relevance of this ORF remains to be determined; such dORFs are present in most IBV strains and other avian gammacoronaviruses, but it is lacking in M41-CK (as is the TRS-B). A recent report has described the same sgmRNA (initiating at the identical TRS) as a novel noncoding RNA of IBV (45). The S gene TRS-B, proposed earlier (34), was also identified.

RiboSeq analysis, in conjunction with RNASeq, revealed that the N protein is not more efficiently translated than other structural proteins, despite being a structural component of IBV virions. It is possible that N expression may be regulated by a putative uORF whose initiation codon is located some 50 nt upstream of the N AUG codon (Fig. 6).

The efficiency of PRF at the IBV ORF1a/ORF1b overlap in natural infection was found to be 33 to 40%. This range is in close agreement with previous *in vitro* measurements of IBV frameshifting efficiency carried out using reporter constructs (5) and is consistent with the notion that the PRF signals of coronaviruses are among the more efficient examples of canonical eukaryotic −1 PRF signals that have been studied to date (25, 46). Whether the modest difference in –1 PRF efficiency measured for Beau-R and M41-CK has biological significance is uncertain, and it may represent experimental variation. The frameshift signal of M41-CK differs from that of Beau-R in only 3 of 81 nucleotide positions, all of which are located in loop 3 of the stimulatory pseudoknot and not expected to affect pseudoknot function or stability (47). As also described for MHV-infected cells (25), there was no evidence that the frameshift-stimulatory pseudoknot induced ribosomal pausing on the slippery sequence. Thus, pausing may not be a component of the frameshifting mechanism, or the pause may be too short-lived to be visualized by the profiling technique.

A meta-analysis of host genes revealed highly specific phasing of the RiboSeq data sets, enabling the accurate determination of the reading frame of translation for individual RPFs. Good phasing in the data sets and substantial read depth also allowed us to examine the translation of viral accessory ORFs. It was evident that both 4b and 4c are efficiently translated at levels comparable to those of the 5a/5b accessory protein-encoding genes. The mechanism by which ribosomes might access ORF4c, however, is not clear. Given the absence of AUG codons within the regions between the 5′ ends of ORF4b and ORF4c in both Beau-R and M41-CK (see Fig. S9 in the supplemental material) and the weak initiation context of the ORF4b start codon, it is possible that a proportion of ribosomes might bypass the ORF4b initiation codon and instead translate ORF4c via leaky scanning (48), although it should be noted that intervening AUG codons do exist in some other IBV strains (Fig. S9).

The relevance to virus gene expression of the sites of significant ribosomal pausing identified in the genome remains to be investigated experimentally. Two of these pause sites appear to correspond to uORFs initiated at non-AUG initiation codons, one in the 5′ UTR and one upstream of the N gene. In each case, ribosomes initiating on the main ORF AUG (ORF1a and N, respectively) could potentiate initiation on the uORFs through stacking of scanning ribosomes, and this could be artefactually increased by the cycloheximide pretreatment used during sample preparation. Two of the other pause sites correspond to ribosomes paused postinitiation early in the coding regions of the S and M genes. A biological explanation for this is lacking at present. We are aware that the treatment of Saccharomyces cerevisiae and Schizosaccharomyces pombe yeast cells with cycloheximide can lead to an early block in elongation in stressed cells (49, 50), but meta-analysis of host genes in our infected cells did not reveal an obvious elongation block. Further, the S and M genes showed deep ribosome coverage along their lengths, inconsistent with a block in elongation. The remaining pause site (in fact, a doublet) appeared during translation of the nsP4 region of the polyprotein close to the C terminus of nsP4. Coronaviral nsP4 proteins are important for the membrane rearrangements required for viral RNA synthesis and contain multiple membrane-spanning domains (42). A possible explanation for the ribosomal pauses seen here is that translation is paused to permit the correct folding of nsP4 into membranes.

In general, the pauses that we discerned during translation of the IBV genome were discrete, substantial in terms of read counts, and reproducible. As mentioned above, their origin is uncertain, but it seems unrelated to the identity of the P-site tRNA. Recent studies have shown that P-site prolyl-tRNA is a strong determinant of ribosomal pausing, partly due to the low rate of peptide bond formation with this amino acid (51, 52). However, none of the stall sites identified here have proline tRNA in the P site. It is possible that the nascent peptide engenders pausing through interactions with the ribosome exit tunnel or chaperones, as clusters of positively charged amino acid residues have been documented to induce ribosome pausing (52, 53). However, such clusters are not evident at the IBV pause sites documented here. The recent developments of methodologies and algorithms to identify and characterize ribosomal pause sites may clarify the situation in future (54, 55).

Our data indicate that the host response to IBV is mediated primarily at the level of transcription with the upregulation of hundreds of genes, many of which have immune-related functions. Changes in translational efficiency were modest, with more genes showing decreased rather than increased translation in response to IBV infection. Many of the transcriptionally upregulated genes identified reflect the host response to virus infection, as seen previously with IBV infection of chickens (56, 57) and with other RNA viruses (58). Some of the protein pathways identified have not previously been associated with coronavirus infection and warrant experimental follow-up, for example, the potential downregulation of transcription of genes linked to FAM20C, a kinase that generates the majority of the extracellular phosphoproteome (59). Also of interest is the translational upregulation of ribosomal protein synthesis in infected cells for both Beau-R and M41-CK. The basis of the attenuated phenotype of Beau-R is not fully understood (reviewed in reference 60) but could potentially involve differential host cell binding (61) or features of the replicase genes (62). The direct comparison of DEGs in M41-CK- and Beau-R-infected primary chick kidney cells here did not identify any preeminent pathways that might reflect their differential pathogenesis, although several cytokines were expressed at a lower level in M41-CK-infected cells (interleukin 6 [IL-6], IL-8L1, beta interferon) (Fig. S11). Overall, these data contribute toward a substantially improved understanding of the early innate immune response to IBV infection, including distinct features of the transcriptional and translational responses.

## MATERIALS AND METHODS

### Virus and cells.

The apathogenic molecular clone of IBV, Beau-R, has been described previously (63). The pathogenic isolate M41-CK (GenBank accession number MK728875.1) has been described previously (64). The two strains have an average nucleotide identity of 93% (assessed in a 1-kb window with a step size of 200 nt). Most of the variation between them occurs as single nucleotide polymorphisms; however, a single large (185-nt) region is present in the 3′ untranslated region (UTR) of Beau-R but is absent from the genome of M41-CK. The Beau-R sequence is identical to the Beau-R sequence (GenBank accession number AJ311317.1), except for two point mutations, one in nsp16 (C19666U; Ser to Leu) and one in N (A27087G, synonymous). Primary chick kidney (CK) cells were produced from 2- to 3 week-old specific-pathogen-free (SPF) Rhode Island Red chickens (65). The chickens were housed in the Home Office-licensed animal facilities of The Pirbright Institute. All animal experimental protocols were approved by the Animal Welfare and Ethical Review Board and performed in accordance with the UK Home Office guidelines and under license for experiments involving regulated procedures on animals protected under the UK Animals (Scientific Procedures) Act 1986. CK cells (0.8 × 10^6^ cells/ml) were plated in 10-cm dishes and upon reaching 100% confluence (at 2 days postseeding) were washed once with phosphate-buffered saline (PBS) and infected with 9.6 × 10^6^ PFU of Beau-R or M41-CK (MOI = ∼3). After 1 h of incubation at 37°C in 5% CO_2_, the inoculum was removed and replaced with 10 ml fresh 1× BES [1× minimal essential Eagle’s medium (MEM), 0.3% tryptose phosphate broth, 0.2% bovine serum albumin, 20 mM *N*,*N*-bis(2-hydroxyethyl)-2-aminoethanesulfonic acid (BES), 0.21% sodium bicarbonate, 2 mM l-glutamine, 250 U/ml nystatin, 100 U/ml penicillin, and 100 U/ml streptomycin]. Cells were harvested at 24 h postinfection when clear regions of cytopathic effect (CPE) were visible.

### Drug treatment, cell harvesting and lysis.

Cycloheximide (CHX; Sigma-Aldrich) was added directly to the growth medium (to 100 μg/ml), and the cells were incubated for 2 min at 37°C before rinsing with 5 ml of ice-cold PBS containing CHX (100 μg/ml). Subsequently, the dishes were incubated on ice and 400 μl of lysis buffer (20 mM Tris-HCl, pH 7.5, 150 mM NaCl, 5 mM MgCl_2_, 1 mM dithiothreitol, 1% Triton X-100, 100 μg/ml cycloheximide, 25 U/ml Turbo DNase [Life Technologies]) was dripped onto the cells. The cells were scraped extensively to ensure lysis, collected, and triturated with a 26-gauge needle 10 times. The lysates were clarified by centrifugation for 20 min at 13,000 × *g* at 4°C, and the supernatants were recovered and stored at −80°C.

### Ribosomal profiling and RNASeq.

Cell lysates were subjected to RiboSeq and RNASeq. The methodologies employed were based on the original protocols of Ingolia and colleagues (23, 66), except that rRNA contamination was removed using a commercial RiboZero Gold magnetic kit (Illumina) and library amplicons were constructed using a small RNA cloning strategy (67) adapted to the Illumina small RNA library preparation kit (v2) to allow multiplexing. The methods used were as described by Chung et al. (68), except that the 5′ and 3′ adaptors included seven consecutive randomized bases at the 3′ and 5′ ends, respectively. This facilitated removal of reads duplicated during PCR amplification of cDNA libraries (69) and reduced ligation bias. The amplicon libraries were deep sequenced using an Illumina NextSeq platform (Department of Pathology, University of Cambridge).

### Computational analysis of RiboSeq and RNASeq data.

Adaptor sequences were trimmed using a FASTX-Toolkit (hannonlab.cshl.edu/fastx_toolkit), and reads shorter than 25 nt following adaptor trimming were discarded. Mapping was performed using the Bowtie (v1) program (70) with parameters -v 2 –best (i.e., maximum 2 mismatches, report best match). Adaptor-trimmed, deduplicated reads were mapped sequentially to host (Gallus gallus) rRNA, IBV Beau-R (GenBank accession number NC_001451.1) or IBV M41-CK (GenBank accession number DQ834384.1) gRNA, Ensembl host noncoding RNA (ncRNA), NCBI RefSeq host mRNA, and the host genome. The order of mapping was tested to check that virus-derived reads were not lost accidentally due to mismapping to host RNA, or vice versa. When performing analyses of viral and host gene expression, only 28- and 29-nt RiboSeq reads (corresponding to RPFs mapping primarily in phase 0) and only ≥40-nt RNASeq reads were used. A 12-nt offset was applied to the 5′ mapping positions of RPFs, to approximate the P-site position of the ribosome (see Fig. S4 in the supplemental material and reference 25). To normalize for different library sizes, reads per million mapped reads (RPM) values were calculated using the sum of total virus RNA plus total host RefSeq mRNA (positive-sense reads only) as the denominator.

Host mRNA RiboSeq and RNASeq phasing distributions were derived from reads mapping internally to the coding regions of ORFs; specifically, the 5′ end of the read had to map between the first nucleotide of the initiation codon and 30 nt 5′ of the last nucleotide of the termination codon, thus, in general, excluding RPFs of initiating or terminating ribosomes. Histograms of the 5′ end positions of the host mRNA reads relative to the initiation and termination codons (Fig. S4 to S7) were derived from reads mapping to RefSeq mRNAs with annotated CDSs at least 450 nt in length and annotated 5′ and 3′ UTRs at least 60 nt in length. When calculating the translation efficiencies of viral genes, only in-phase (i.e., phase 0 with respect to the ORF in question) RiboSeq reads were counted.

For host differential expression analyses, nonribosomal, nonviral reads in each library were mapped to the Gallus gallus (v5.0) assembly (December 2015) using the STAR aligner (71), with gene annotations being from Ensembl release 94 (72). A maximum of two mismatches were allowed when mapping. Read counts per gene (protein-coding genes only) were obtained using the HTSeq framework (73), with a requirement that reads map entirely within the forward strand coding sequence (htseq-count parameters, -m intersection-strict -s yes -t CDS). For each comparison of experimental groups, only genes with an average of at least 50 mapped reads were included in the differential expression analyses. GO term enrichment analysis was carried out using the topGO package in R (74), and Fisher’s exact test was used to assess the enrichment of individual GO terms in specific gene lists. Protein-protein interaction networks were constructed using the Search Tool for Retrieval of Interacting Genes (STRING) database (44).

### Data availability.

Sequencing data have been deposited in ArrayExpress (http://www.ebi.ac.uk/arrayexpress) under the accession number E-MTAB-7849.

## Supplementary Material

Supplemental file 1

Supplemental file 2

Supplemental file 3

Supplemental file 4
