# Supplementary material for: Comparative Analysis of Gene Expression in Virulent and Attenuated Strains of Infectious Bronchitis Virus at Subcodon Resolution
Source: J Virol. 2019 Aug 28;93(18):e00714-19. doi: 10.1128/JVI.00714-19 (PMC6714804; doi:10.1128/JVI.00714-19)

Type	Condition	Total	De-duplicated	viral gRNA fwd	viral gRNA rev	host mRNA
**Repeat 1**						
RiboSeq	Beau-R-infected	55,648,104	32,461,160	1,470,253	630	3,070,823
	M41-CK-infected	47,459,412	24,416,659	1,838,613	792	4,193,904
	Mock-infected	46,640,167	26,170,072	399	1	9,400,391
RNASeq	Beau-R-infected	27,720,370	12,171,506	2,365,784	4,842	882,421
	M41-CK-infected	24,939,168	14,070,349	1,829,331	4,070	1,616,990
	Mock-infected	25,036,626	15,462,396	2,737	9	2,037,543
**Repeat 2**						
RiboSeq	Beau-R-infected	55,392,595	30,089,677	778,394	191	3,017,330
	M41-CK-infected	51,319,886	27,664,513	540,016	63	1,978,766
	Mock-infected	56,813,327	26,535,892	159	1	3,413,589
RNASeq	Beau-R-infected	26,792,717	15,637,095	2,606,240	8,245	1,840,748
	M41-CK-infected	46,163,533	2,680,836	106,741	388	127,579
	Mock-infected	26,095,361	15,802,166	4,589	8	1,920,949

Table S1. Library composition statistics: table of read counts per library. Random 7nt sequences were added to the ends of both 5′ and 3′ adaptors during library preparation to facilitate the removal of duplicate reads introduced during PCR. All libraries were de-duplicated following the removal of the adaptor sequences, as described in the Materials and Methods section. Numbers of reads mapped to the forward (fwd) and reverse (rev) strands of the viral gRNA are shown separately in each case; only forward strand-mapping reads are included in the host mRNA counts.

TRS	Genomic TRS sequence	No. reads rep. 1	No. reads rep. 2	% rep. 1	% rep. 2
S [U3]	AAAA**CU**G**AACAA**AAGA	176	158	2.58	1.53
S [G3]	AAAA**CU**G**AACAA**AAGA	129	120	1.89	1.16
S (int.)	CAAA**CUUA**C**CAA**ACAA	15	12	0.22	0.12
3/E [U3]	GUAA**CU**G**AACAA**UACA	93	97	1.36	0.94
3/E [G3]	GUAA**CU**G**AACAA**UACA	140	125	2.05	1.21
M	AAAA**CUUAACAA**UCCG	571	569	8.36	5.50
4a/4b	ACUGG**U**G**A**C**CAA**AGCG	109	147	1.60	1.42
5_1	UUUA**CUUAACAA**AAAC	81	189	1.19	1.83
5_2	AAAA**CUUAACAA**AUAC	1,085	2,237	15.88	21.63
N	CUUU**CUUAACAA**AGCA	4,376	6,643	64.05	64.24
dORF	UUUGAG**UAACAU**AAUG	57	44	0.83	0.43

Table S2. (A) Chimeric TRS-spanning reads identified in IBV Beau-R samples. Residues matching the leader TRS (TRS-L) are shown in bold.

TRS	Genomic TRS sequence	No. reads rep. 1	No. reads rep. 2	% rep. 1	% rep. 2
S [U3]	AAAA**CU**G**AACAA**AAGA	200	9	5.21	6.34
S [G3]	AAAA**CU**G**AACAA**AAGA	250	12	6.51	8.45
S (int.)	CAAA**CUUA**C**CAA**ACAC	1	0	0.03	0
3/E [U3]	GUAA**CUUAACAA**UACA	182	2	4.74	1.41
3/E [G3]	GUAA**CUUAACAA**UACA	0	0	0	0
M	AAAA**CUUAACAA**UCCG	877	46	22.83	32.39
4a/4b	ACUGG**U**G**A**C**CAA**AGCG	303	11	7.89	7.75
5_2	AGCG**CUUAA**U**AA**AUAC	464	8	12.08	5.63
N	CUUU**CUUAACAA**AGCA	1565	54	40.74	38.03

Table S2. (B) Chimeric TRS-spanning reads identified in IBV M41-CK samples. Residues matching the leader TRS (TRS-L) are shown in bold.

ORF	Beau-R_infected_1	Beau-R_infected_2	M41-CK_infected_1	M41-CK_infected_2
M	136602	83269	88020	62575
N	79944	57353	78482	67917
5a	32588	24129	18069	14804
3a	23678	17306	23009	15819
E	20575	14600	16785	14046
5b	17318	6468	16061	5844
3b	12074	7330	9163	6778
4b	8962	7932	8957	5466
S	5527	3708	13273	9578
4c	2069	652	2852	1289
1a	141	117	114	80
1b	67	42	81	53

Table S3. RiboSeq reads (RPKM) for individual coding sequences. Note, the 1b values include some reads that likely derive from ribosome-protected fragments (RPFs) at the S ORF overlap, where a large peak contains some reads in the same phase as the 1b ORF. In the ribosomal frameshifting calculations of Figure 3, reads in overlapping regions were excluded from the calculations.

GO.ID	Term	Annotated	Significant	Expected	P-value
GO:0010469	regulation of signaling receptor activity	53	7	0.58	1.2×10^{-6}
GO:0048661	positive regulation of smooth muscle cell proliferation	18	5	0.2	3.5×10^{-5}
GO:2000352	negative regulation of endothelial cell apoptotic process	9	3	0.1	9.7×10^{-5}
GO:0060585	positive regulation of prostaglandin-endoperoxide	2	2	0.02	1.2×10^{-4}
GO:0033141	positive regulation of peptidyl-serine phosphorylation of ST...	2	2	0.02	1.2×10^{-4}
GO:0014826	vein smooth muscle contraction	2	2	0.02	1.2×10^{-4}
GO:1900625	positive regulation of monocyte aggregation	2	2	0.02	1.2×10^{-4}
GO:0014824	artery smooth muscle contraction	3	2	0.03	3.5×10^{-4}
GO:0003100	regulation of systemic arterial blood pressure by endothelin	3	2	0.03	3.5×10^{-4}
GO:1901842	negative regulation of high voltage-gated calcium channel ac...	3	2	0.03	3.5×10^{-4}
GO:0006955	immune response	244	12	2.66	5.9×10^{-4}
GO:1904754	positive regulation of vascular associated smooth muscle cel...	4	2	0.04	6.9×10^{-4}
GO:0071356	cellular response to tumor necrosis factor	40	4	0.44	8.4×10^{-4}
GO:0043154	negative regulation of cysteine-type endopeptidase activity ...	18	3	0.2	8.8×10^{-4}
GO:0001516	prostaglandin biosynthetic process	8	3	0.09	1.09×10^{-3}
GO:0071498	cellular response to fluid shear stress	8	3	0.09	1.09×10^{-3}
GO:0002931	response to ischemia	5	2	0.05	1.14×10^{-3}
GO:0090051	negative regulation of cell migration involved in sprouting ...	5	2	0.05	1.14×10^{-3}
GO:0019229	regulation of vasoconstriction	5	2	0.05	1.14×10^{-3}
GO:0070373	negative regulation of ERK1 and ERK2 cascade	22	3	0.24	1.16×10^{-3}

Table S4. Top 20 most significantly enriched GO terms among genes which are down-regulated in IBV M41-CK-infected cells relative to IBV Beau-R-infected cells. Column "Annotated" shows the total number of genes in the data set which are members of that GO term; "Significant" shows the number that are significantly differentially expressed; and "Expected" shows the proportion of genes that would be expected to occur in a random sample.

GO.ID	Term	Annotated	Significant	Expected	P-value
GO:0042026	protein refolding	7	2	0.01	4.4×10^{-5}
GO:0051085	chaperone cofactor-dependent protein refolding	9	2	0.01	7.6×10^{-5}
GO:0034605	cellular response to heat	17	2	0.03	2.9×10^{-4}
GO:0090074	negative regulation of protein homodimerization acti	1	1	0	1.6×10^{-3}
GO:1905719	protein localization to perinuclear region of cytoplas	1	1	0	1.6×10^{-3}
GO:1903895	negative regulation of IRE1-mediated unfolded prote respon...	1	1	0	1.6×10^{-3}
GO:0070194	synaptonemal complex disassembly	1	1	0	1.6×10^{-3}
GO:0006880	intracellular sequestering of iron ion	1	1	0	1.6×10^{-3}
GO:0034620	cellular response to unfolded protein	36	3	0.06	2.5×10^{-3}
GO:1902949	positive regulation of tau-protein kinase activity	2	1	0	3.2×10^{-3}
GO:1903071	positive regulation of ER-associated ubiquitin-depen pro...	2	1	0	3.19×10^{-3}
GO:1901896	positive regulation of calcium-transporting ATPase activity	2	1	0	3.19×10^{-3}
GO:0090084	negative regulation of inclusion body assembly	3	1	0	4.78×10^{-3}
GO:1905323	telomerase holoenzyme complex assembly	3	1	0	4.78×10^{-3}
GO:0044130	negative regulation of growth of symbiont in host	3	1	0	4.78×10^{-3}
GO:0007141	male meiosis I	3	1	0	4.78×10^{-3}
GO:0031396	regulation of protein ubiquitination	73	2	0.12	5.31×10^{-3}
GO:0035973	aggrephagy	4	1	0.01	6.38×10^{-3}
GO:0061635	regulation of protein complex stability	4	1	0.01	6.38×10^{-3}
GO:1900273	positive regulation of long-term synaptic potentiatioı	4	1	0.01	6.38×10^{-3}

Table S5. Top 20 most significantly enriched GO terms among genes which are up-regulated in IBV M41-CK-infected cells relative to IBV Beau-R-infected cells. Column "Annotated" shows the total number of genes in the data set which are members of that GO term; "Significant" shows the number that are significantly differentially expressed; and "Expected" shows the proportion of genes that would be expected to occur in a random sample.

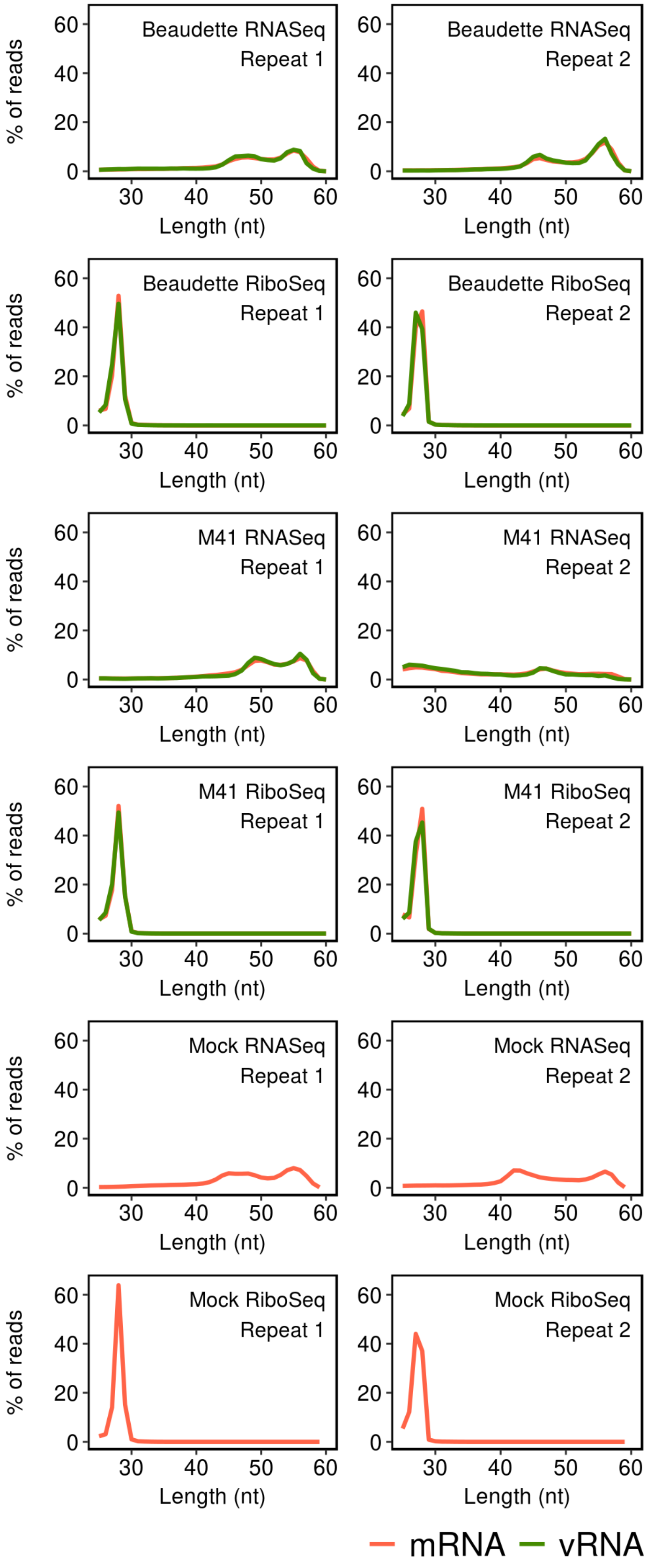

Figure S1. Length distribution of reads mapped to the internal regions of protein-coding sequences on viral RNA (vRNA; green lines) and host mRNA (red lines).

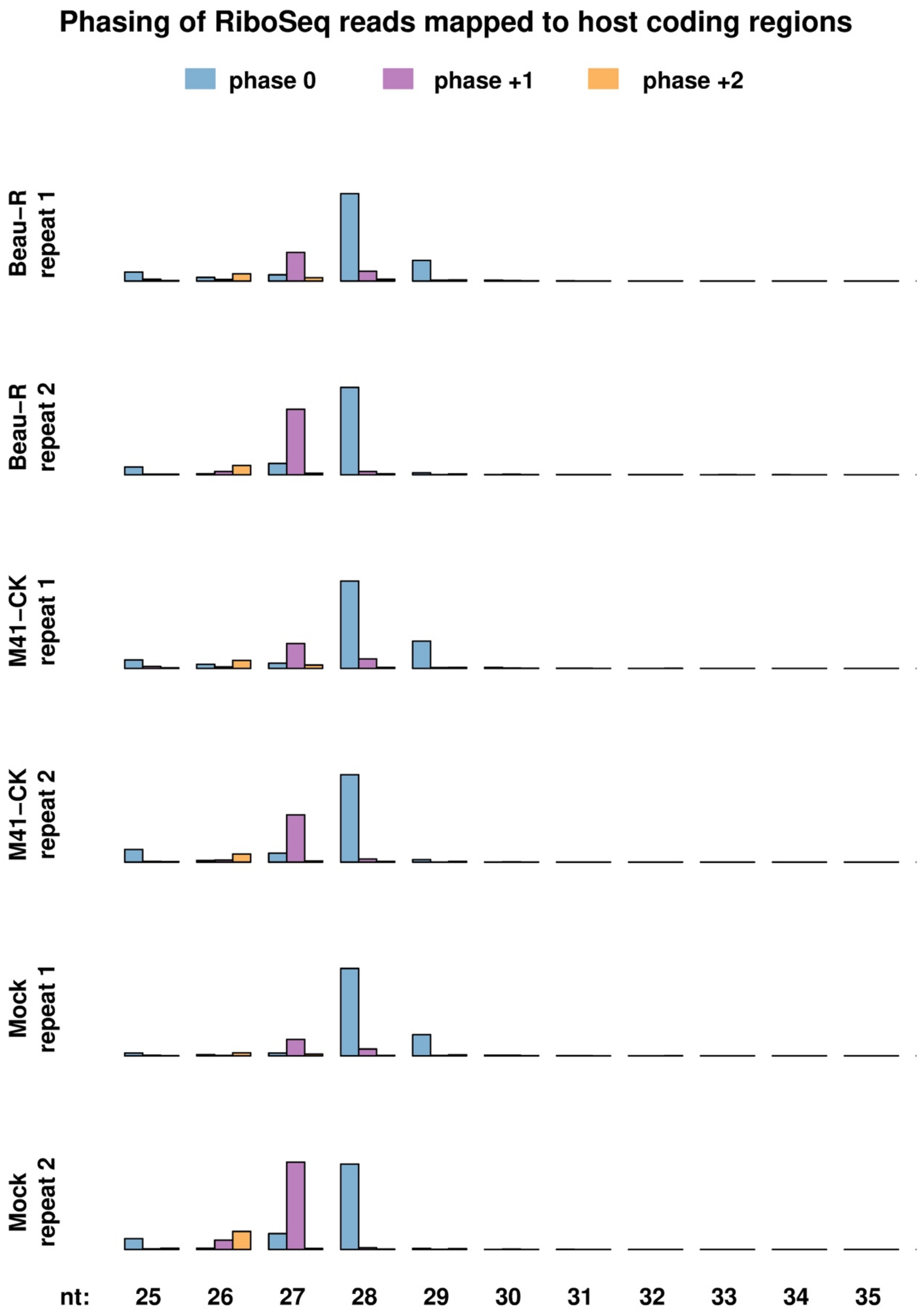

Figure S2. Phasing of RiboSeq reads mapped to the internal regions of protein-coding sequences in host mRNAs for different RPF lengths.

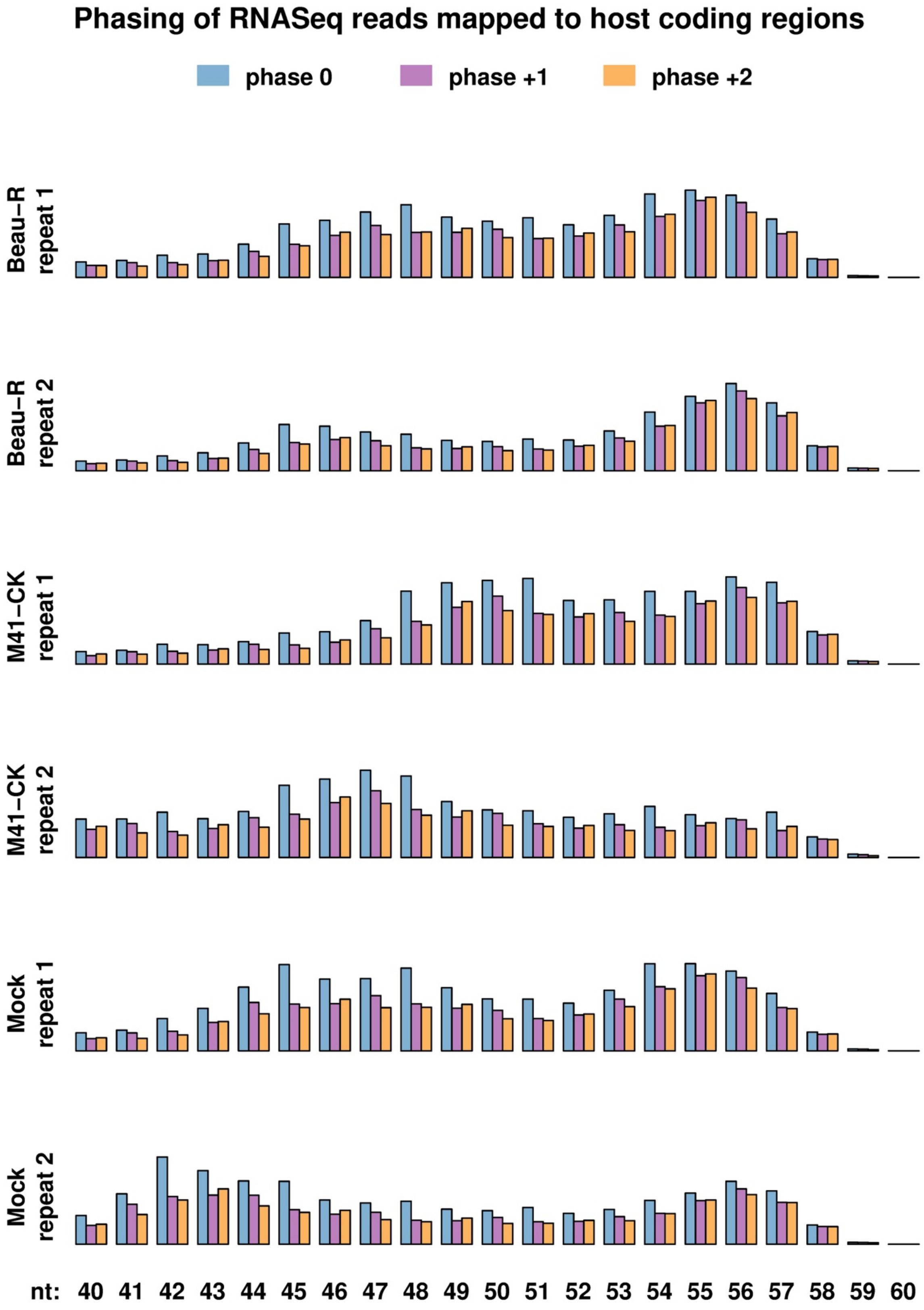

Figure S3. Phasing of RNASeq reads mapped to the internal regions of protein coding sequences in host mRNAs. To aid visualisation, only reads between 40 nt and 60 nt in length are shown.

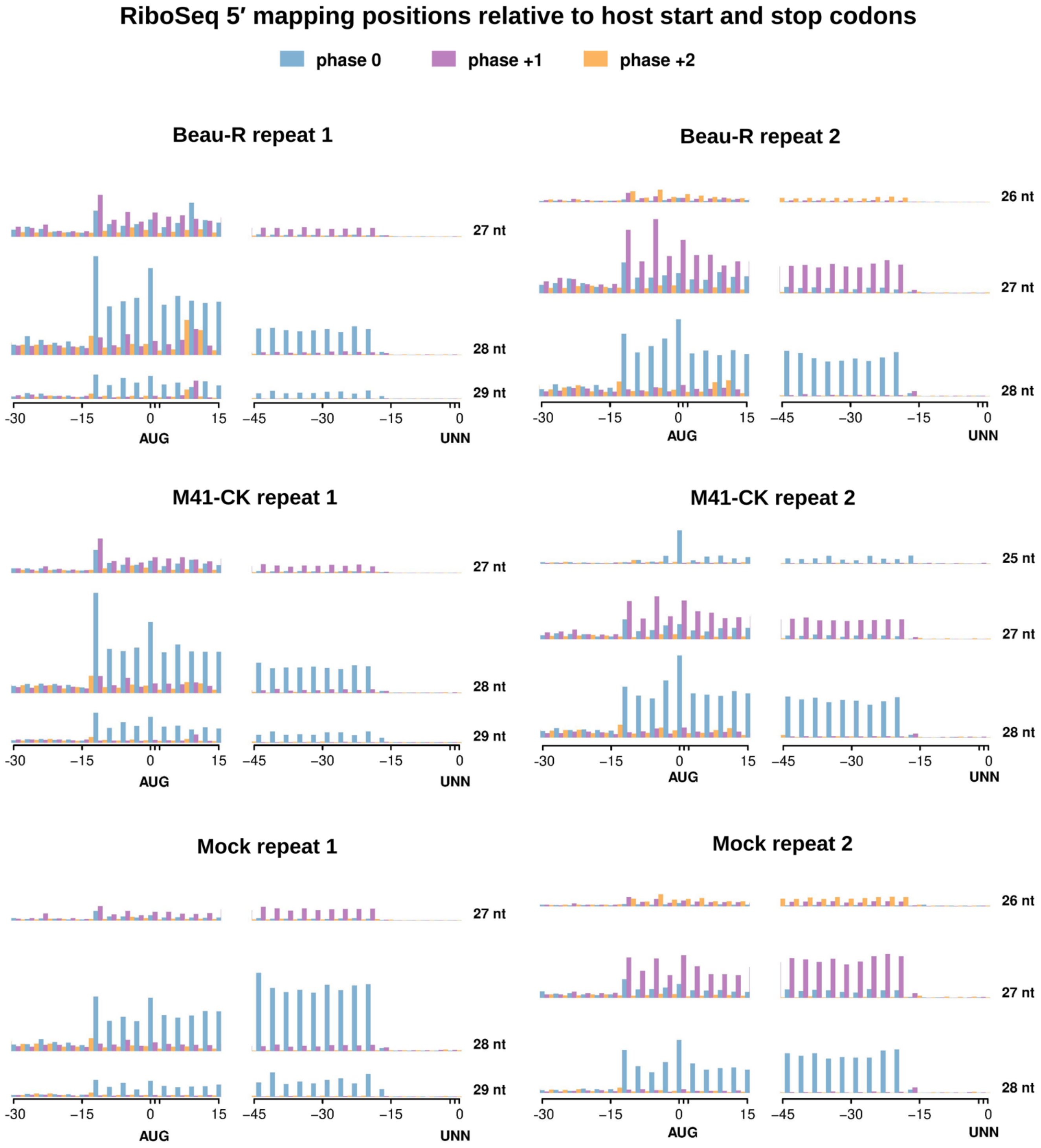

Figure S4. Histograms of 5′ mapping positions of RiboSeq reads relative to host mRNA start (AUG) and stop (UNN) codons. Positions indicated are relative to the first nt of the AUG codon (left) and the last nt of the UNN codon (right). The three most abundant read lengths are plotted for each library.

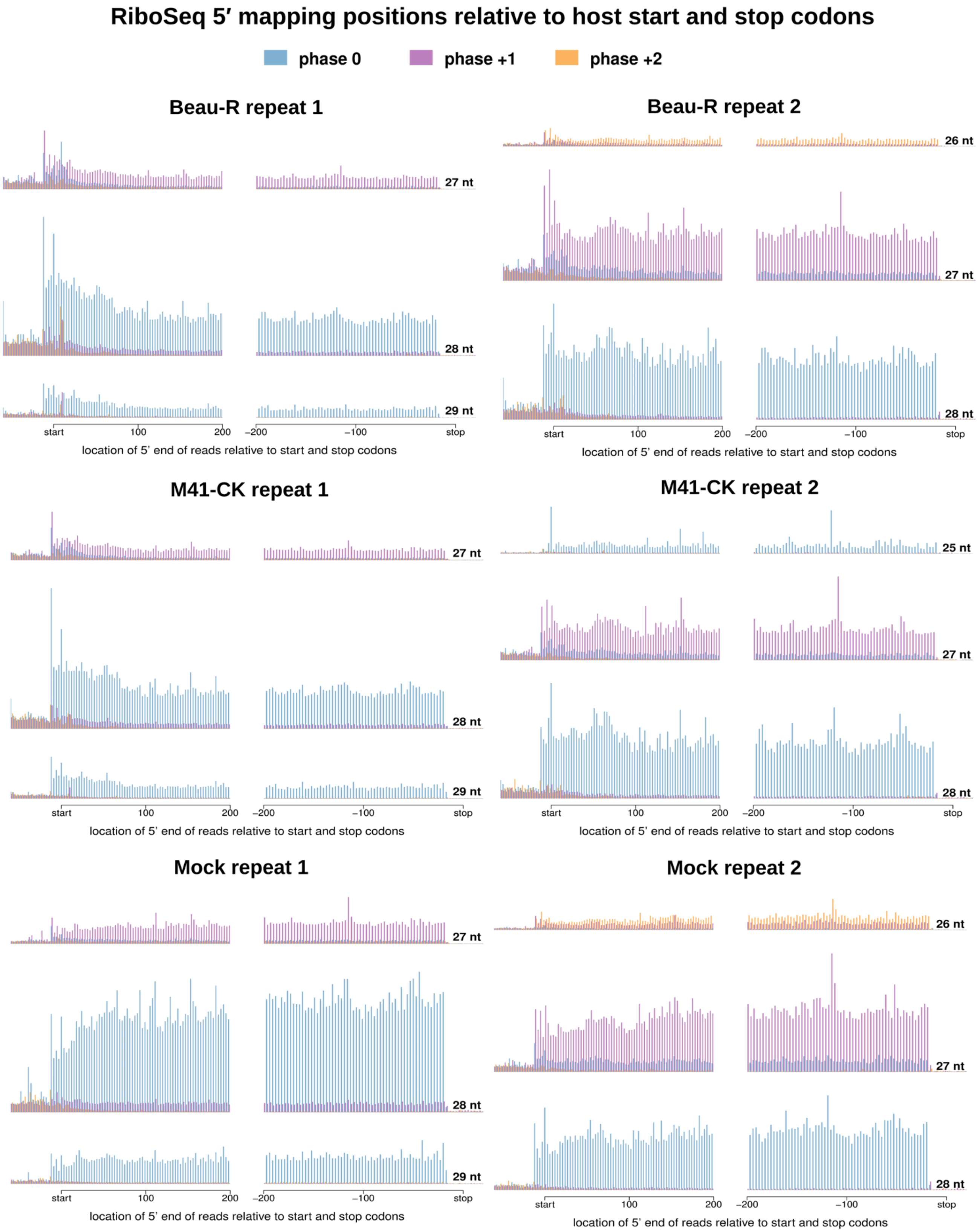

Figure S5. Histograms of 5′ mapping positions of RiboSeq reads relative to host mRNA start and stop codons. The three most abundant read lengths are plotted for each library.

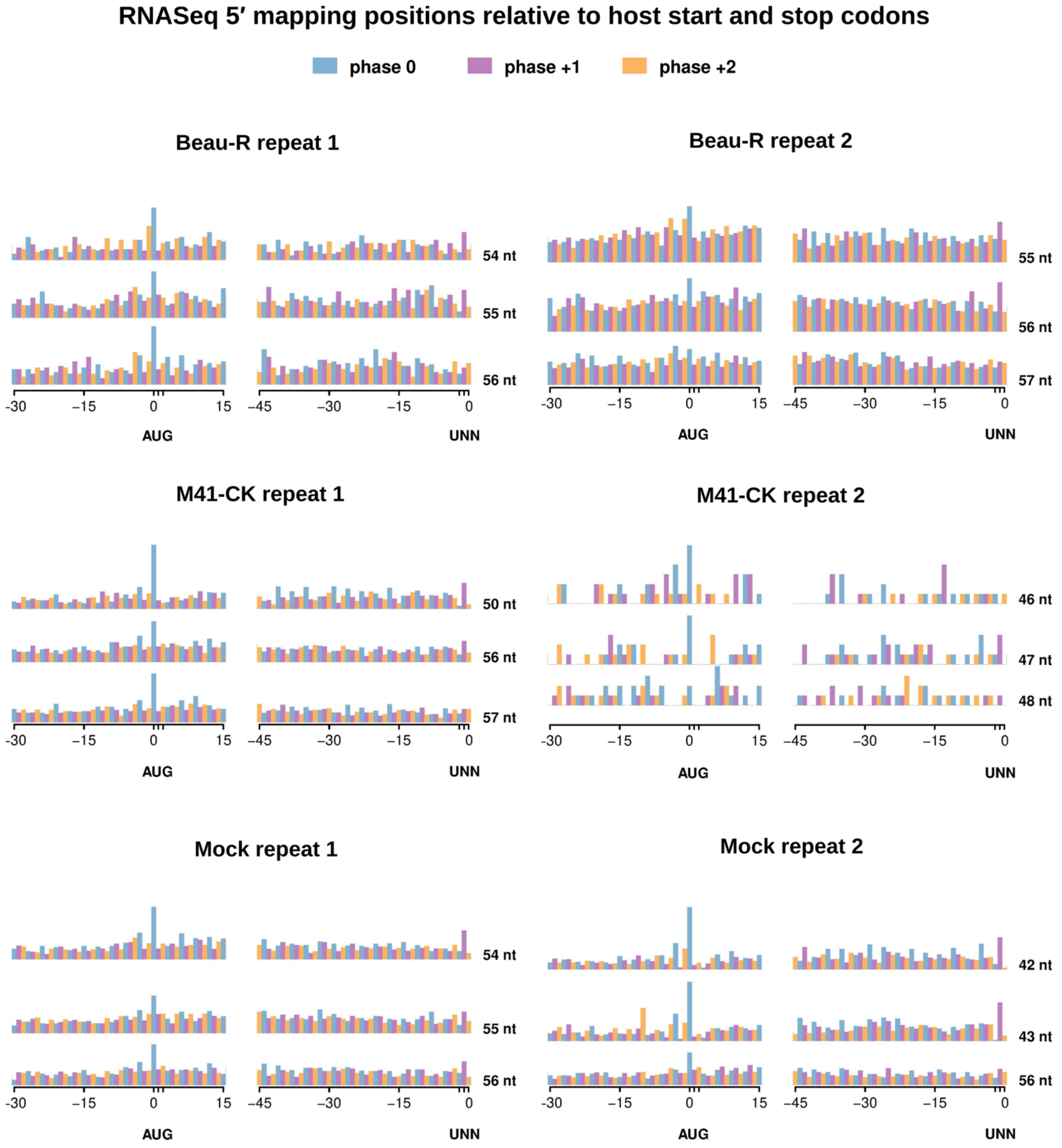

Figure S6. Histograms of 5′ mapping positions of RNASeq reads relative to host mRNA start (AUG) and stop (UNN) codons. Positions indicated are relative to the first nt of the AUG codon (left) and the last nt of the UNN codon (right). The three most abundant read lengths are plotted for each library.

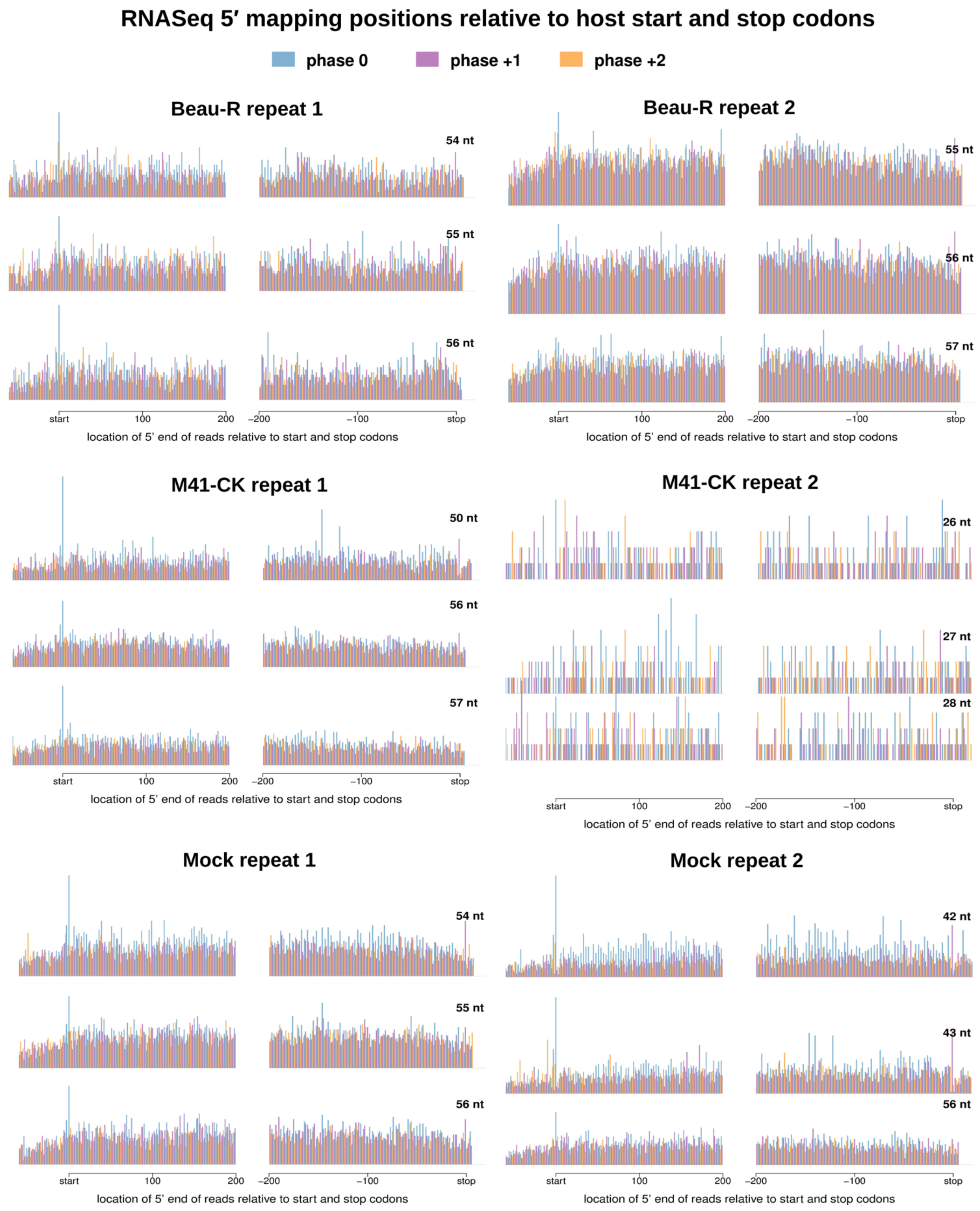

Figure S7. Histograms of 5′ mapping positions of RNASeq reads relative to host mRNA start and stop codons. The three most abundant read lengths are plotted for each library.

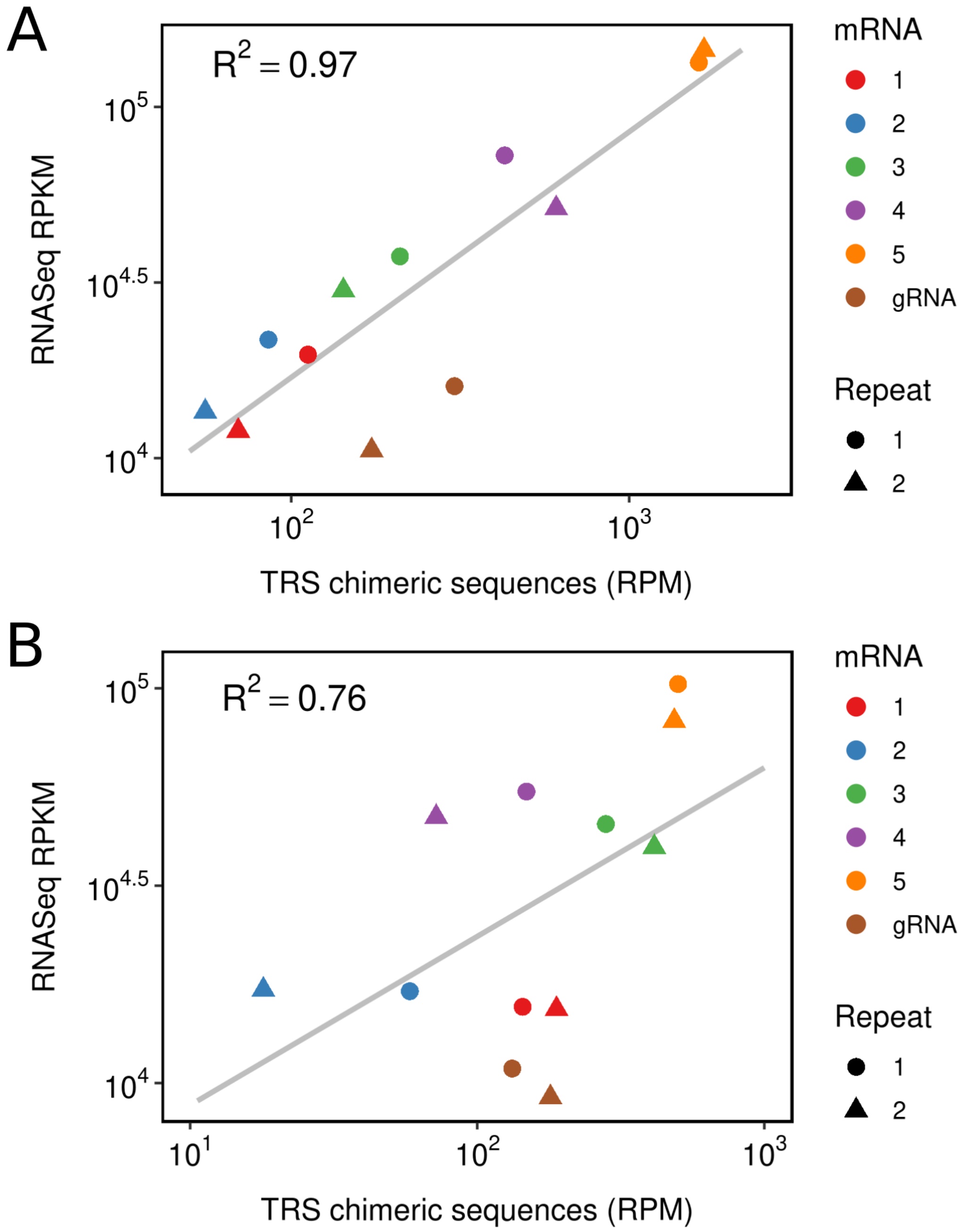

Figure S8. Relative abundances of sgmRNAs (sgmRNA 1 to 5) and full-length gRNA for **(A)** IBV Beau-R and **(B)** IBV M41-CK, as measured by decumulating RNASeq coverage (see Methods) or by counting chimeric reads spanning the TRS sequence. RNASeq densities are expressed as reads per kilobase per million mapped reads (RPKM), and chimeric TRS-spanning reads are expressed as reads per million mapped reads (RPM).

```
IBV B1648      CUUUACACAUAAAUGUGUGUGUAGAGAGUAUUUAAGAUUAUUCUUUGACAGUGCCUCU
IBV Beaudette  CUUUACACAUAAAUGUGUGUGUAGAGAGUAUUUAAAAUUAUUCUUUAAUAGCGCCUCU
IBV M41        CUUUACACAUAAAUGUGUGUGUAGAGAGUAUUUAAGACUAUUCUUUAAUAGUGCCUCU
               ************************************* * ******** * ** ******

IBV B1648      AUUUUAAGAGCGCGGAAGAGUAUUAUUUUUGAGGAUAUUAAUAUAAAUCCUCUUUGUUUC
IBV Beaudette  GUUUUAAGAGCGCAUAAGAGUAUUUAUUUUGAGGAUACUAAUAUAAAUCCUCUUUGUUUU
IBV M41        AUUUUAAGAGCGCAUACGAGUAUUUAUUUUGAGGAUAUUAAUAUAAAUCCUCUUUGUUUU
                ************  * *******  *********** *********************

IBV B1648      AUACUCUCCUUUCAGGAGUUAUUAUUUAAAAAACAGUUUUUCCACUCUUUUGUGCCAAAA
IBV Beaudette  AUACUCUCCUUUCAAGAGCUA---------------------------------------
IBV M41        AUACUCUCUUUUCAAGAGCUAUUAUUUAAAAAACAGUUUUUCCACUCUUUUGUGCCAAAA
               ******** ***** *** **

IBV B1648      ACUAUUGUUGUCAAUGGUGUAACCUUUCAAGUAGAUAAUGGAAAAGUCUACUACGAAGGA
IBV Beaudette  ----------UUAACGGUGUUACCUUUCAAGUAGAUAAUGGAAAAGUCUACUACGAAGGA
IBV M41        ACUAUUGUUGUUAACGGUGUUACCUUUCAAGUGGAUAAUGGAAAAGUCUACUACGAAGGA
                         * ** ***** *********** ***************************
```

Figure S9. Alignment of IBV sequences, showing the position of the ORF4b and ORF4c initiation codons (green). A 49-nt deletion in the Beau-R strain prematurely truncates the ORF4b gene by bringing a UAG codon (red) in frame. Note the absence of AUG codons between the starts of ORF4b and ORF4c in both IBV Beau-R and IBV M41-CK, consistent with a leaky scanning mechanism for 4c expression. Conversely, the genome of the Belgian nephropathogenic strain B1648 contains an intervening AUG (underlined; 2-codon ORF) towards the 3′ end of ORF4b.

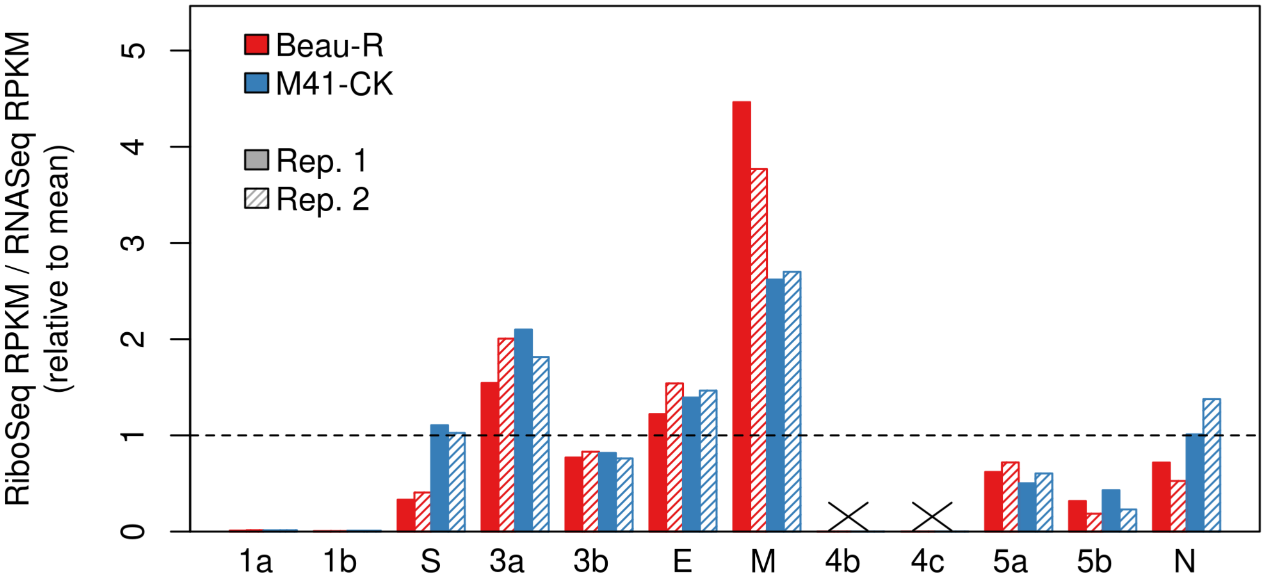

Figure S10. Viral gene translation efficiency values calculated using decumulated RNASeq reads (expressed as reads per kilobase per million mapped reads [RPKM]). The ratio of RiboSeq RPKM to RNASeq RPKM is plotted relative to the mean across all samples. TE values for 4b and 4c could not be quantified (see Main text).

Figure S11. STRING analysis of the relationship between differentially expressed genes in comparisons of IBV Beau-R, IBV M41-CK and mock-infected chick kidney cells (**panels A to I**). The network nodes represent the proteins encoded by the differentially expressed genes. Seven different coloured lines link a number of nodes and represent seven types of evidence used in predicting associations. A red line indicates the presence of fusion evidence; a green line represents neighborhood evidence; a blue line represents co-ocurrence evidence; a purple line represents experimental evidence; a yellow line represents text-mining evidence; a light blue line represents database evidence; and a black line represents co-expression evidence.

(**A**) Beau-R vs Mock downregulated transcripts

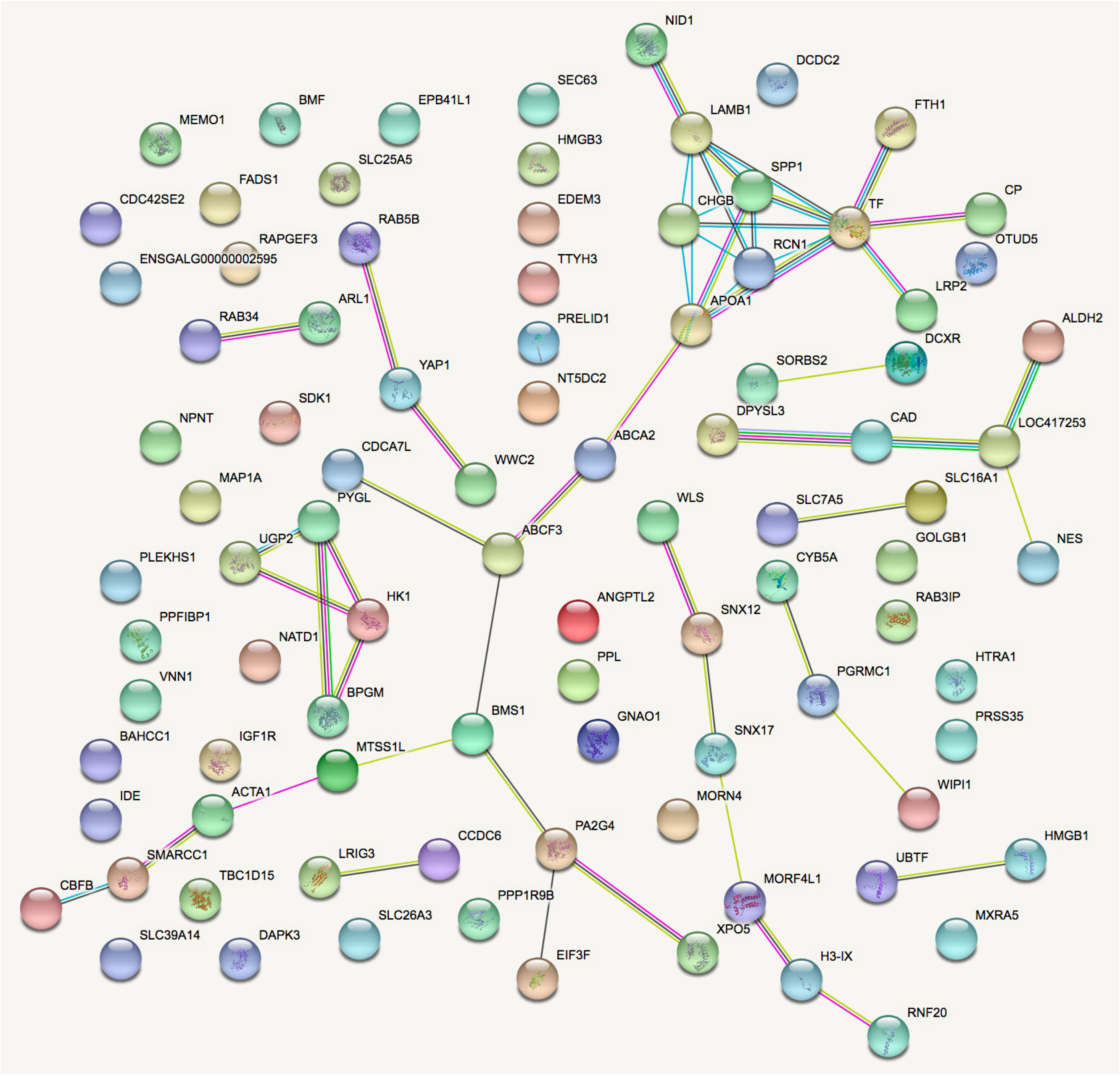

(**B**) M41-CK vs Mock downregulated transcripts

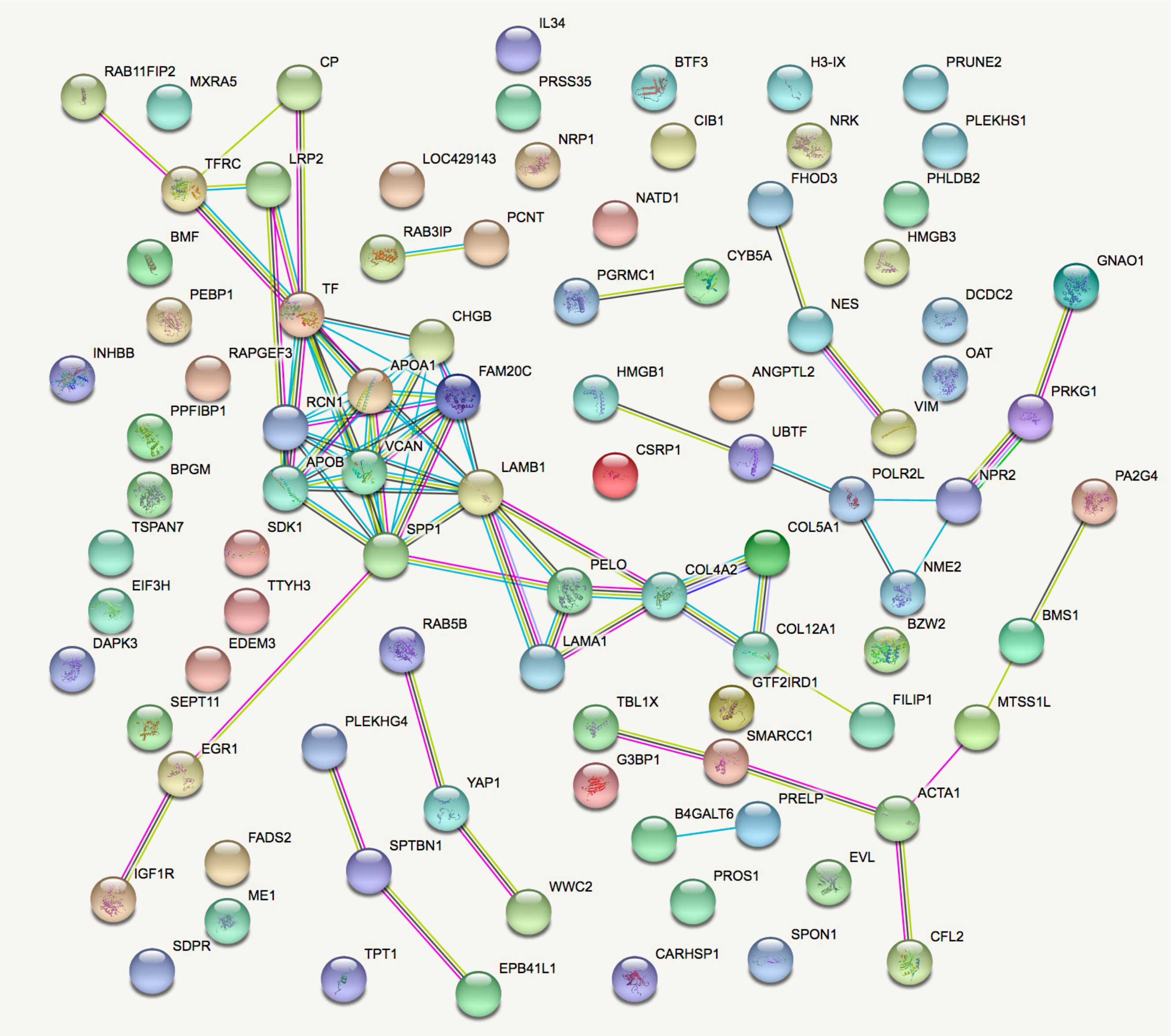

(**C**) Beau-R vs Mock upregulated transcripts

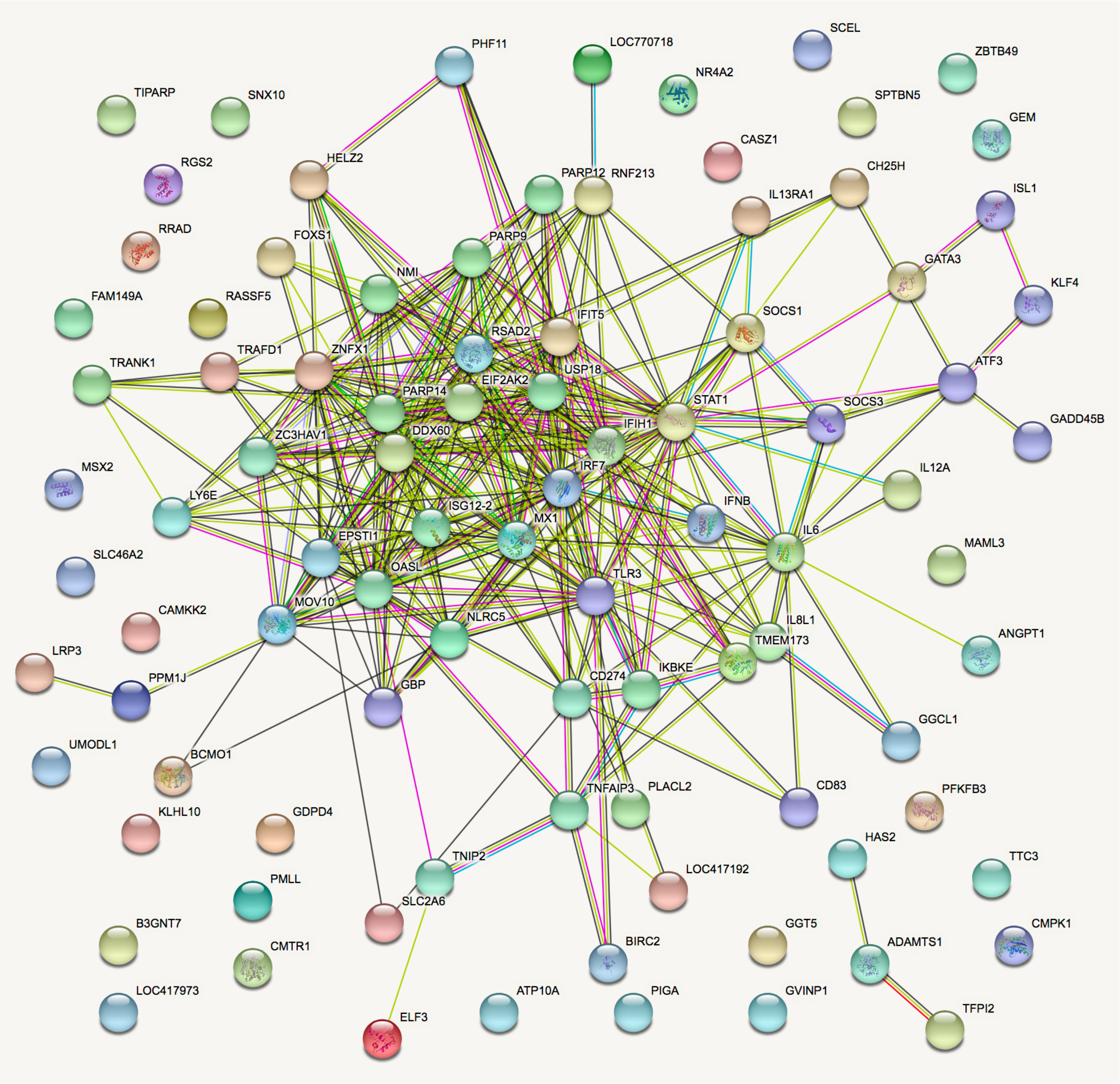

(**D**) M41-CK vs Mock upregulated transcripts

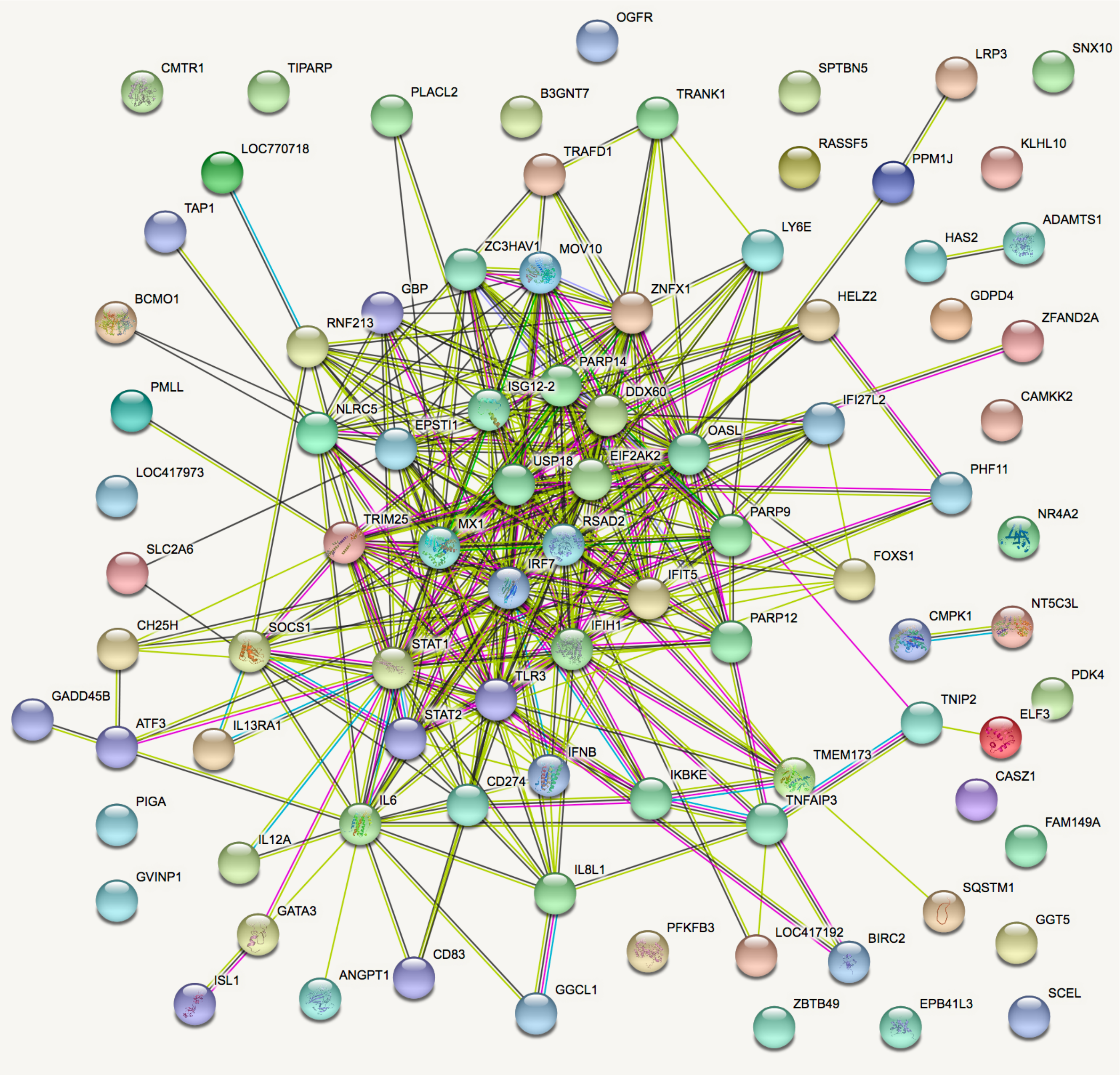

(**E**) Beau-R vs Mock downregulated translation

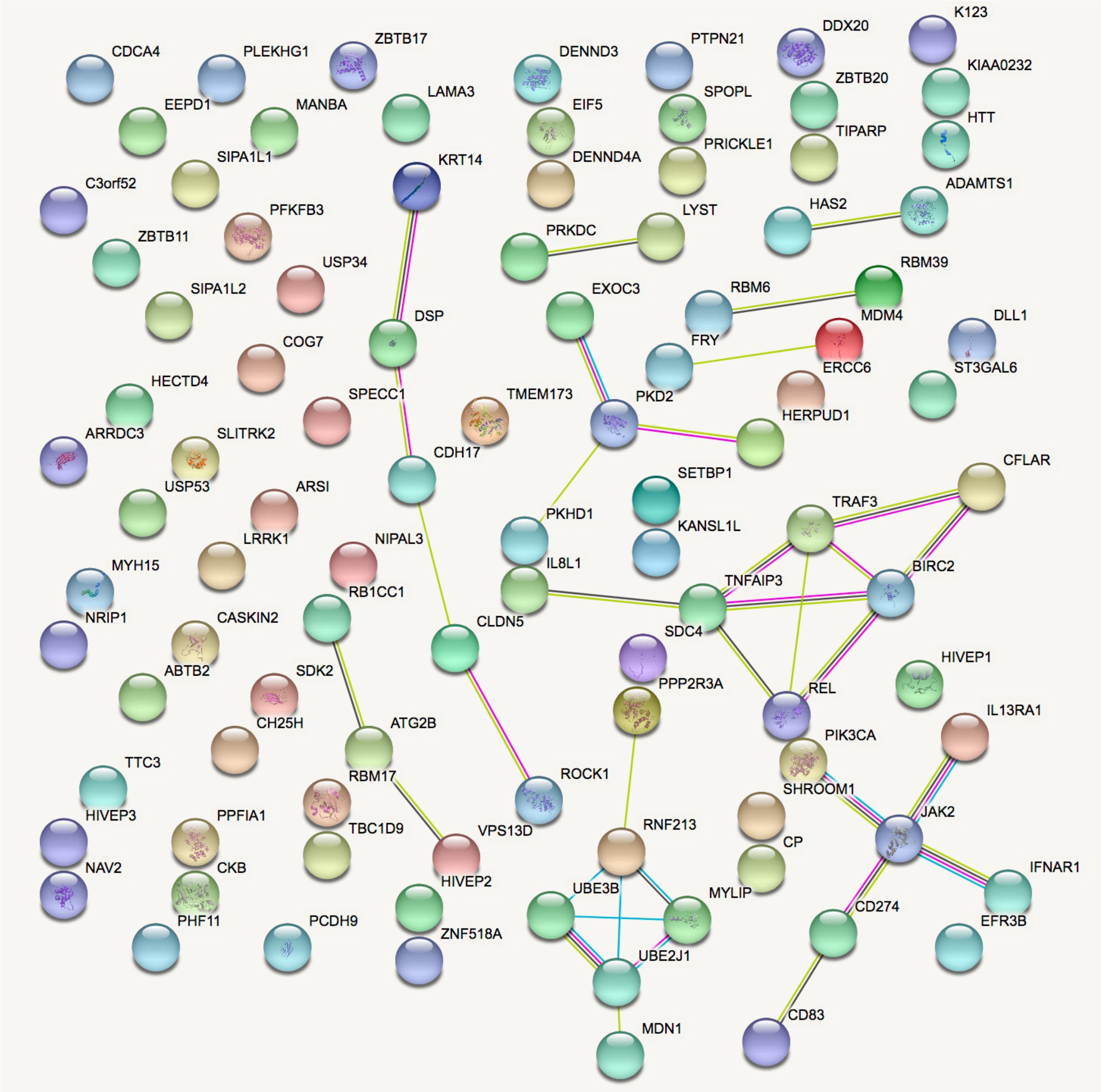

(**F**) M41-CK vs Mock downregulated translation

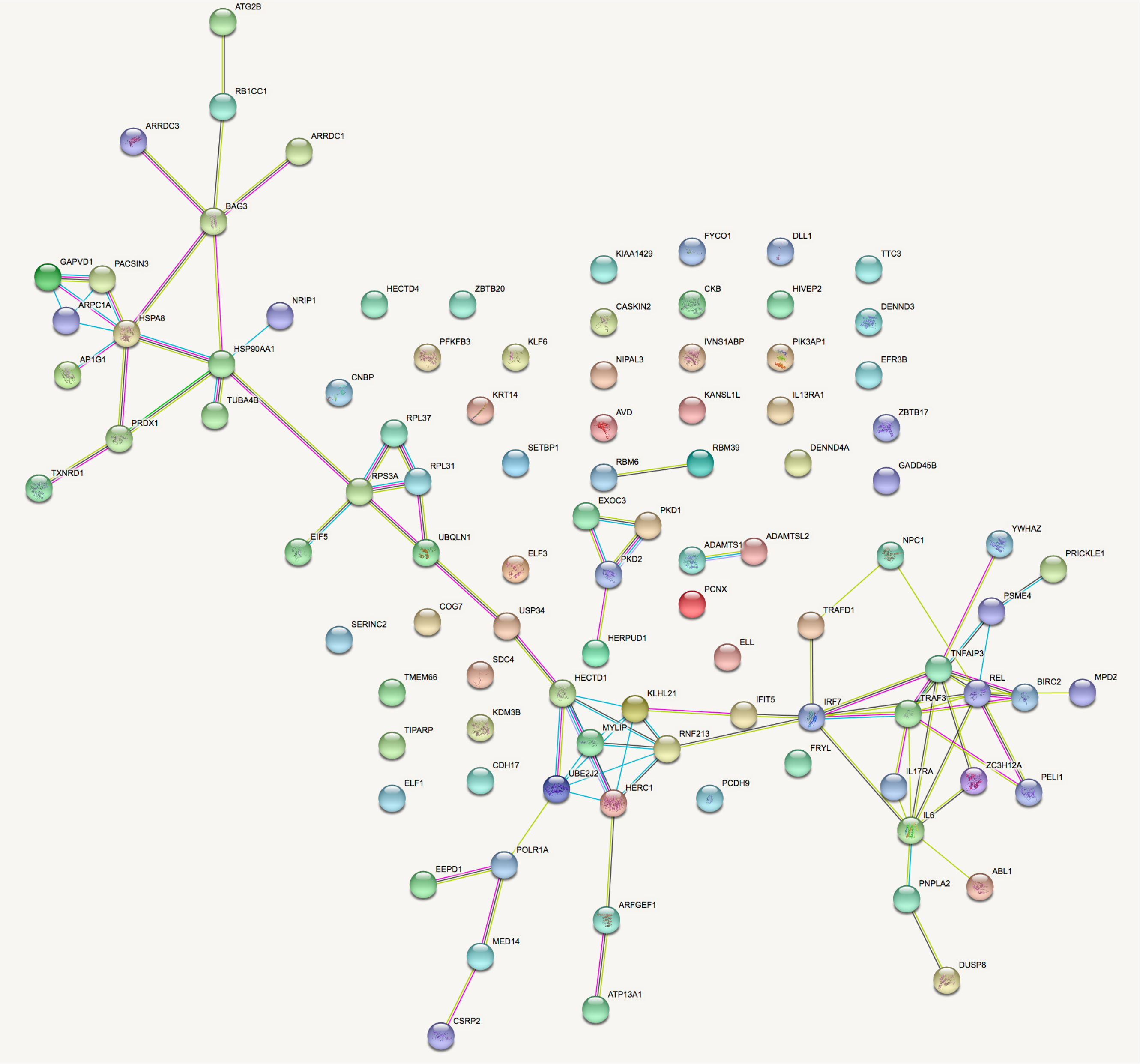

(**G**) Beau-R vs Mock upregulated translation

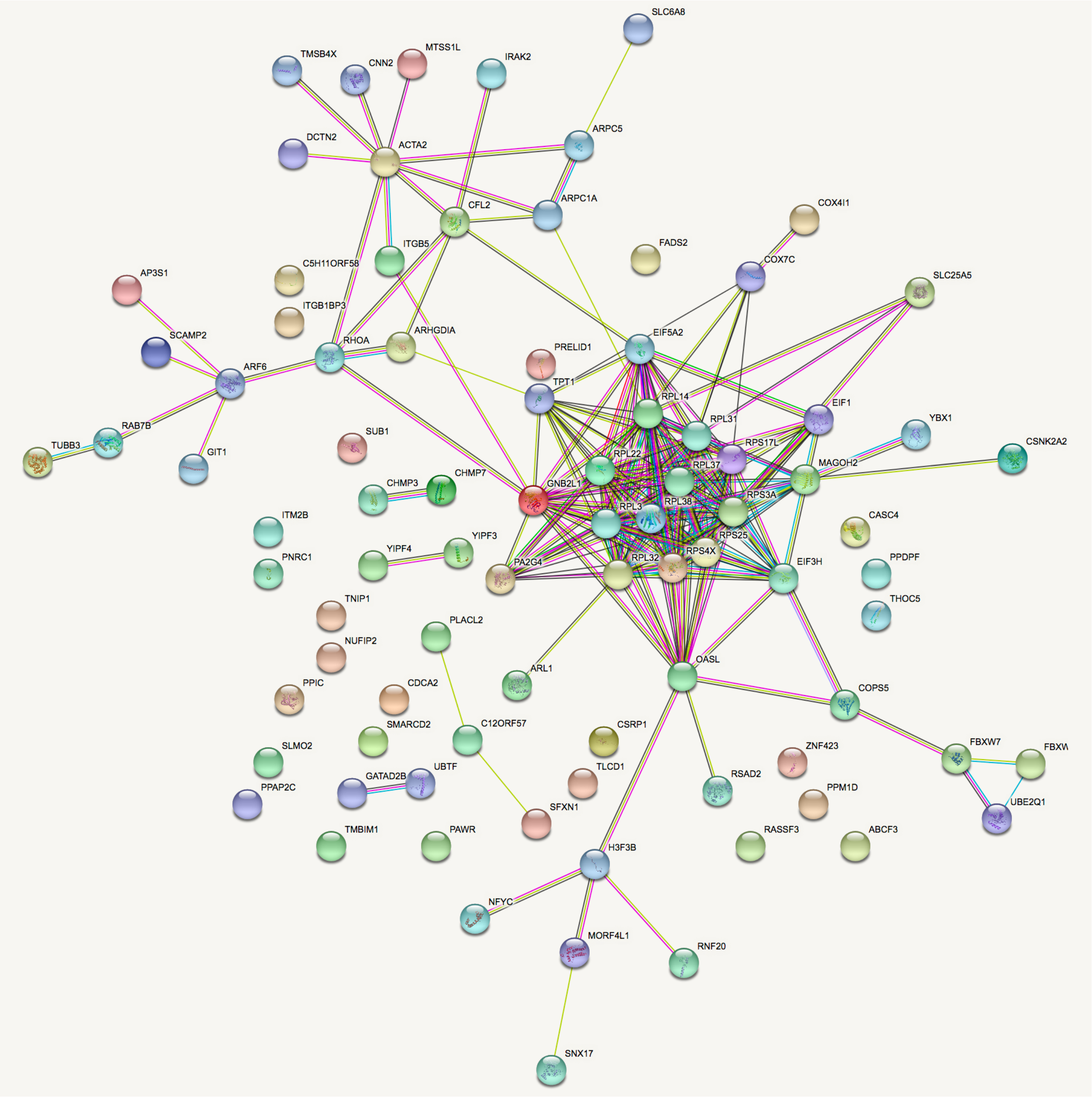

(**H**) M41-CK vs Mock upregulated translation

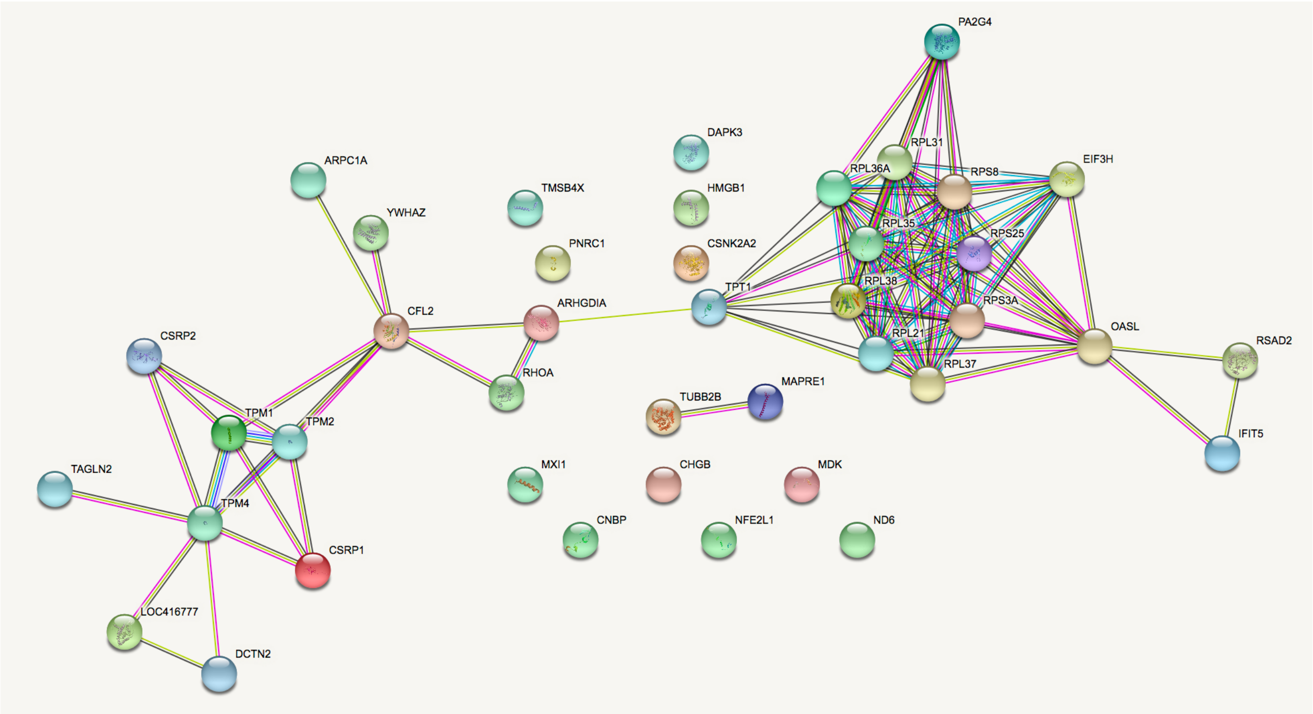

(**I**) M41-CK vs Beau-R downregulated transcripts

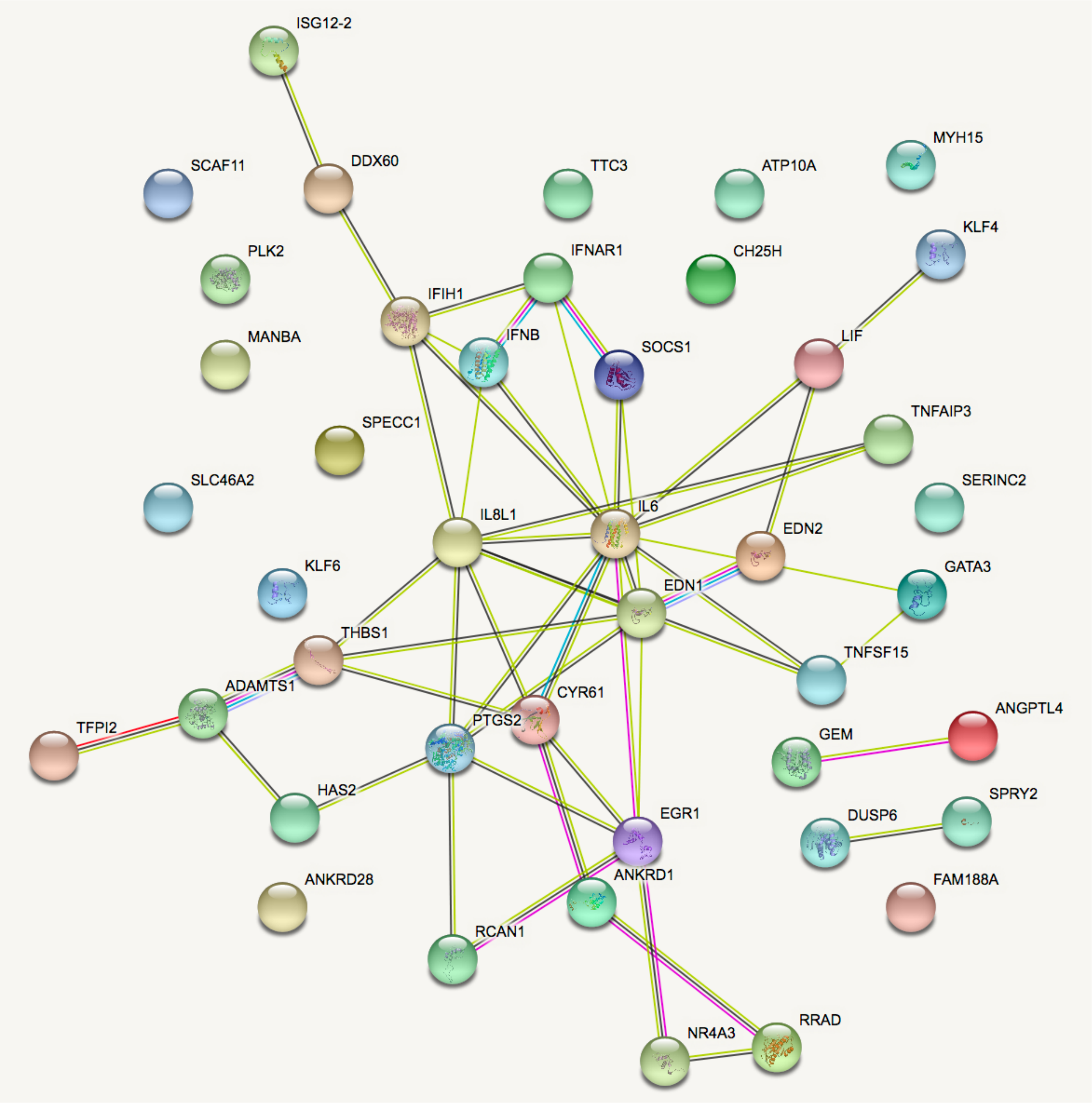

Supplement: Supplemental file 4 [file JVI.00714-19-s0004.pdf]
